# Comprehensive battery aging dataset: capacity and impedance fade measurements of a lithium-ion NMC/C-SiO cell

**DOI:** 10.1038/s41597-024-03831-x

**Published:** 2024-09-16

**Authors:** Matthias Luh, Thomas Blank

**Affiliations:** Karlsruhe Institute of Technology (KIT), Institute for Data Processing and Electronics (IPE), Eggenstein-Leopoldshafen, 76344 Germany

**Keywords:** Batteries, Batteries, Energy, Electrical and electronic engineering

## Abstract

Battery degradation is critical to the cost-effectiveness and usability of battery-powered products. Aging studies help to better understand and model degradation and to optimize the operating strategy. Nevertheless, there are only a few comprehensive and freely available aging datasets for these applications. To our knowledge, the dataset^1^ presented in the following is one of the largest published to date. It contains over 3 billion data points from 228 commercial NMC/C+SiO lithium-ion cells aged for more than a year under a wide range of operating conditions. We investigate calendar and cyclic aging and also apply different driving cycles to cells. The dataset^1^ includes result data (such as the remaining usable capacity or impedance measured in check-ups) and raw data (i.e., measurement logs with two-second resolution). The data can be used in a wide range of applications, for example, to model battery degradation, gain insight into lithium plating, optimize operating strategies, or test battery impedance or state estimation algorithms using machine learning or Kalman filtering.

## Background & Summary

Batteries are vital for storing electrical energy in portable devices, electric vehicles (EVs), and electricity grids powered by a high share of renewable energy. In EVs and stationary energy storage systems, the cost and lifetime of the battery are critical factors for the economic viability and usability of the product. The performance of battery cells diminishes over time. This is manifested by a loss of capacity and an increase in electrical impedance. In many studies that consider the cost of battery degradation, the lifetime of a battery is estimated using simple assumptions about the lifetime and number of usable cycles. However, the aging type and rate strongly depend on operating conditions, such as the operating temperature, charging rate, and State of Charge (SoC) window. An in-depth understanding of the aging mechanisms and dependencies of the cells is vital to maximizing the lifetime of the battery through an appropriate operating strategy. This can not only save costs but also material resources.

Battery aging can be represented, for example, by (semi-)empirical, electrochemical/physics-based, or machine-learning-assisted / statistical / data-driven models^2,3^. In order to derive, calibrate, or train these models, measurement data from real battery cells is beneficial or even required. The dependencies of the aging mechanisms are manifold. For instance, solid electrolyte interphase (SEI) growth mainly depends on the SoC and temperature of the cell. The SEI continues to grow over time even if the cell is not in use. Therefore, it is often classified as “calendar aging”. On the other hand, lithium plating depends on the SoC, temperature, charging rate, and age of the cell^4–7^. It may take place while the cell is charged, particularly at cold temperatures, high C-rates, and high SoC. At a certain stage, it can rapidly degrade the cell even under moderate charging conditions^7^. Since lithium plating only occurs when the cell is cycled, it is an example for an aging mechanism associated with the “cyclic aging” mode. If all of these dependencies and their typically nonlinear behavior should be considered in the degradation model, the dataset used for modeling has to be as extensive and diverse as possible since test conditions varying all of these dependencies should be included.

However, most battery aging datasets published in academia are relatively small since only a few operating conditions are tested due to cost or other resource constraints. Datasets collected by cell, battery, or EV manufacturers are mostly confidential and not published at all. Moreover, many publications only present “result data”, e.g., the remaining usable capacity over time collected during check-ups (CUs) / reference performance tests (RPTs). Often, only selected figures showing results are shown, and no reusable data is provided for download and further usage by other researchers and companies. Raw data with higher temporal resolution, e.g., the cell’s voltage, current, and temperature, is rarely provided, even though it can be vital for accurate modeling. Besides, cells are often only aged until the remaining capacity reaches 70 or 80% of the nominal capacity. However, for second-life applications, a much lower capacity threshold would be beneficial to estimate the value of an aged battery and derive a suitable operating strategy in the subsequent application.

The most promising comprehensive battery aging studies we found are summarized in Table 1. The table lists the number of cells examined in the study, their cell chemistry (cathode/anode material), whether capacity (cap.), impedance (imp.), and raw log data are published, if the data and an accompanying publication (pub.) are available open-access (OA), how many test conditions were investigated, and which parameters were varied for the calendar (CAL) and cyclic (CYC) aging conditions. Investigated anode materials are lithium iron phosphate (LFP), nickel manganese cobalt (NMC), nickel cobalt aluminum (NCA), lithium manganese oxide (LMO), or blends of multiple materials. Cathodes may include graphite (C) or graphite with silicon (C-Si). Parameters varied include temperature (T), storage State of Charge (SoC), SoC window and Depth of Discharge (DoD), charge (C_c_), discharge rate (C_d_), general current rate (C_c/d_), charging protocol (CP), pressure (p), and check-up interval (CU).

**Table 1:** Overview of comprehensive battery aging datasets.

Study	Cell count	Cell chemistry	Cap. data	Imp. data	Raw data	OA data	OA pub.	Number of test conditions: variation of parameters
Schimpe *et al*.^19^	111	LFP/C	*✓*	—	—	—	*✓*	• 54x CAL: T, SoC • 30x CYC: T, C_c/d_, CP • 6x current profile: T
Mohtat *et al*.^20,21^	31	NMC/C	*✓*	*✓*	*✓*	*✓*	*✓*	• 27x CYC: T, DoD, C_c_, C_d_, p
*EVERLASTING* project^22,23^	70	NMC/C-Si	*✓*	—	*✓*	*✓*	*✓*	• 12x CAL: T, SoC • 14x CYC: T, C_c_, C_d_ • 9x driving profile: T, profile, DoD
Uddin *et al*.^24^	63	NCA/C	*✓*	*✓*	—	—	*✓*	• 9x CAL: T, SoC • 6x CYC: DoD, SoC_high_, C_d_ • 6x driving profile (dynamic conditions)
*Batteries2020* project^25–30^	158	NMC/C	*✓*	(*✓*)	—	—	*✓*	• 10x CAL: T, SoC • 36x CYC: T, DoD, SoC_mid_, C_c_, C_d_
*MOBICUS* project^31–34^	258	NMC/C, NMC-LMO/C	*✓*	*✓*	—	—	*✓*	• 16x CAL: T, SoC •≥ 17x CYC: T, SoC_mid_, C_c_, C_d_ • 6x mixed aging: T, SoC, C_c_, C_d_, ratio
Naumann *et al*.^35–39^	114	LFP/C	*✓*	*✓*	—	*✓*	—	• 17x CAL: T, SoC • 21x CYC: T, SoC_mid_, DoD, C_c_, C_d_, CP
Wildfeuer *et al*.^9,14^	196	NCA/C-Si	*✓*	*✓*	*✓*	*✓*	—	•≥ 47x CAL: T, SoC, CU •≥72x CYC: T, SoC_mid_, DoD, C_c_, C_d_ •≥68x dynamic/altering/random cond.
This study^1^	228	NMC/C-Si	*✓*	*✓*	*✓*	*✓*	*✓*	• 16x CAL: T, SoC • 48x CYC: T, SoC_min/max_, DoD, C_c_, C_d_ • 12x driving profiles: T, SoC_max_, C_c_, C_d_

Moreover, battery aging data of different cell chemistries collected from various studies and online archives is available on batteryarchive.org. The raw cycling and result data can be visualized and compared online.

Since there are comparatively few freely available and comprehensive raw cell aging datasets that can be used to model battery degradation, we decided to collect a comprehensive dataset^1^ and make it available to the public. In our experiment, 228 cells were aged at 76 different operating conditions to generate a very diverse dataset. 16 calendar aging, 48 cyclic aging, and 12 driving profile parameter sets were selected, using four different temperatures between 0 and 40°C, various SoCs, and charging and discharging rates. Three cells were operated per condition to increase the statistical significance of the results and robustness of the experiment, for example, in case a single cell fails.

**Fig. 1:**
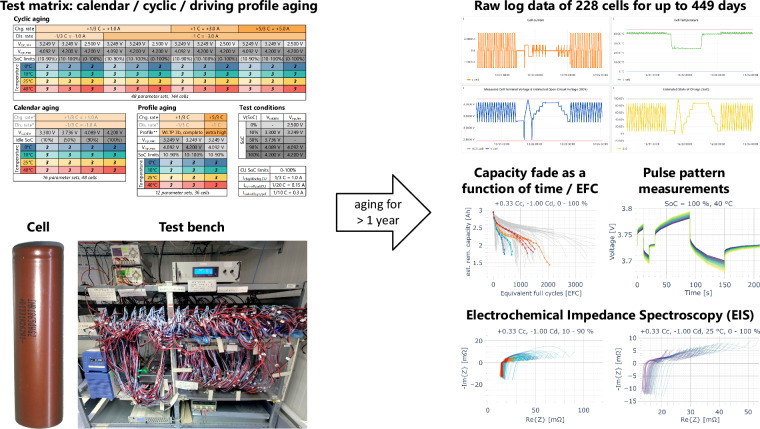
Graphical abstract of the battery degradation study and the generated datasets.

Among others, our dataset^1^ contains the remaining usable capacity and charged/discharged energy during cycling and the CUs, in which all cells are fully charged and discharged at room temperature. The results of an electrochemical impedance spectroscopy (EIS) measurement and a pulse pattern applied at different SoCs at operating and room temperature in the CUs are also included. Unlike in most previously published aging studies, raw log data collected every two seconds during the experiment is also published (compare Fig. 1). For example, it contains the cell voltage, current, temperature, charge, energy, and the estimated SoC and open-circuit voltage (OCV). Since both the result data (remaining capacity and impedance) and raw log data are included, possible usages of the dataset are very versatile. Among others, it is conceivable to use the battery aging dataset to derive degradation models based on semi-empirical or machine-learning approaches or to use the raw cycling data to test and validate SoC or cell impedance estimators.

While many studies only focus on battery degradation until 70 to 80% of the nominal capacity remains, we continue investigating aging until the cells only have 40 to 50% of the nominal capacity. This helps to understand battery degradation after the “knee point”, when the capacity drops significantly faster. In addition, it allows for a better determination of the residual value of an EV battery or the development of optimal operating strategies for Second Life applications.

## Methods

In this chapter, the cell and test parameter selection, the test procedure, the experimental setup, and subsequent data processing will be explained in detail. This is particularly relevant to ensure good reproducibility of the results and to maximize the value of the dataset^1^ for various applications, such as accurate cell aging modeling (compare^6^^, p. 701^).

### Cell selection

The motivation for the study was to estimate and optimize the aging behavior of EV batteries in Vehicle-to-Grid (V2G) operation. Among the most common cell chemistries used in EVs nowadays are NMC, NCA, and LFP batteries^8^. The lifetime of modern LFP cells can exceed the lifetime of typical passenger cars^8^, and thus, the additional use for V2G is a minor concern for cell degradation. However, for NMC and NCA chemistries, optimizing the operating strategy can have a relevant impact on battery aging and its associated costs. As mentioned, a comprehensive aging dataset of NCA cells was recently published by Wildfeuer *et al*.^9^. In contrast, our study focuses on an NMC cell chemistry.

We compared more than 150 lithium-ion cells from eight manufacturers available in 2021 and selected the *LG INR18650HG2* as a suitable, commercially available candidate to represent cells typically used in EVs. Even though cells in EV usually have a higher capacity per cell, a cylindrical model with a smaller nominal capacity was used to decrease the cost and complexity of the experiment. According to the technical information of the cell^10^, its cathode consists of an NMC chemistry, and the anode contains graphite and silicon oxide (SiO). As can be seen in Table 2, the cell features a relatively high energy density, fast charging capability, and wide charging temperature range.

**Table 2:** Properties of the selected cell (LG INR18650HG2)^10,18^ — please refer to the product specification provided by the manufacturer for details about the safe operating conditions allowed.

Form factor	18650 (cylindrical)	Nominal capacity	3000 mAh
Diameter	ca. 18.3 mm	Usable energy	11.0 Wh
Height	ca. 65.0 mm	Nominal voltage	3.60 V
Weight	ca. 46 g	Allowed voltage range	2.0–4.2 V
Anode material	Graphite with SiO	Used voltage range	2.5–4.2 V
Cathode material	LiNi_x_Mn_y_Co_1-x-y_O_2_ (*“H-NMC”*^10^)	Maximal current	6.0 A (charging)
			20.0A (discharging)
Energy density	240 Wh/kg	Temperature range	− 20 to +75°C (operation)
	640 Wh/l		− 5 to +50°C (charging)

### Parameter set selection

Three different operating modes were considered: calendar aging cells, which are not cycled between check-ups; cyclic aging cells, which are continuously charged and discharged; and profile aging cells, which are discharged according to representative driving cycles.

**Fig. 2:**
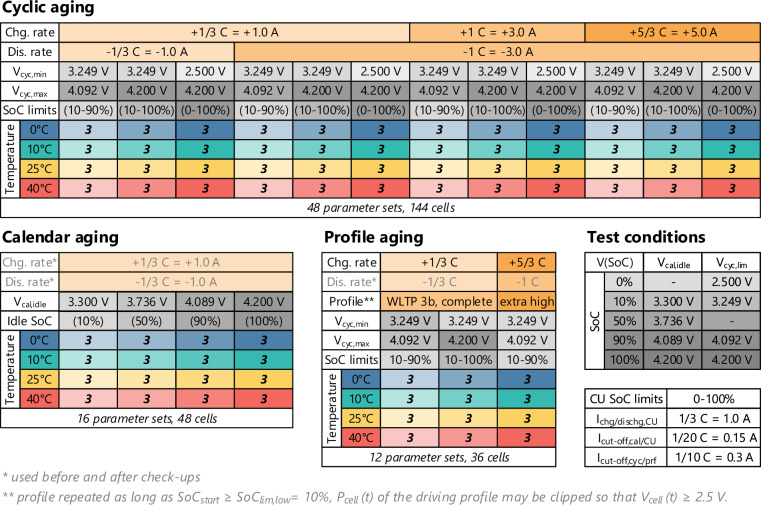
Test matrix for the battery aging study with 76 parameter sets for 228 cells (three cells are used per parameter set).

The test matrix containing all selected operating conditions is summarized in Fig. 2. Further details are included in the “Cycling Experiment Cell Overview” Excel spreadsheet in the published dataset^1^. The parameters were selected as a trade-off between a wide range of operating conditions typical for an electric car battery and a limited number of battery cells. All conditions were conducted at four different operating temperatures (0°C, 10°C, 25°C, 40°C). Three voltage ranges (2.5–4.2 V, 3.249–4.2 V, 3.249–4.092 V, corresponding to SoC ranges of approximately 0–100%, 10–100%, 10–90%) and four charging and discharging rate combinations were selected for cyclic aging. A focus of the study was the effect of different charging rates (+1/3 C, +1 C, +5/3 C) since attaining high charging speeds is usually a limiting factor for EVs and a concern regarding degradation, particularly lithium plating. On the other hand, the cell is specified for significantly higher discharging rates (6.67 C) than normally occurring in EV applications (typically less than 1–2 C), so it is anticipated that the variation of the discharging rate has a subordinate impact on degradation. Only two discharging rates (−1/3 C, −1 C) were investigated to reduce the number of cells in the experiment. Four voltages (3.3 V, 3.736 V, 4.089 V, 4.2 V, corresponding to SoCs of approximately 10%, 50%, 90%, 100%) were used for calendar aging cells. For the profile aging cells, the Worldwide Light-duty Test Cycle (WLTC) for Class 3b vehicles (typical passenger cars) of the Worldwide harmonized Light vehicles Test Procedure (WLTP)^11^ was used as a driving cycle. The applied battery cell power was calculated with the JRC Python Gearshift Calculation Tool^12^ and the assumptions about the EV and its battery listed in Table 3.

**Table 3:** Assumptions to derive the WLTP cycle battery cell power.

Input for the Gearshift Calculation Tool	
v_max_	167 km/h
P_rated_	150 kW
n_rated_	11000 1/min
n_idle_	58 1/min
#g	6
m_test_	1840 kg
f_0_	200 N
f_1_	0.35 N/(km/h)
f_2_	0.032 N/(km/h)^2^
p(n)	min(n*0.04136 kW*min, P_rated_)
ndv	65.868 (1/min)/(km/h)

Calculation of the battery power	
*η*_motor_	90% (constant)
*η*_inverter_	95% (constant)
P_auxiliary_	1 kW (constant)
v_min,recuperation_	8 km/h
P_max,recuperation_	64 kW
Resulting WLTP consumption: 15.8 kWh/100 km	

Calculation of the cell power	
battery capacity	64 kWh
cell capacity	3 Ah
cells in series	96
cells in parallel	60

Two regular use cases, in which the EV charges with 1/3 C to 90% (4.092 V) or 100% (4.2 V), were selected. In a third case, only the “extra high” part of the Class 3b WLTC is applied, and the cell is charged with 5/3 C, representing an intense highway use of the vehicle.

The cyclic and profile aging cells are charged with a constant current (CC), constant voltage (CV) protocol. The cyclic aging cells are also discharged in this manner. During regular operation, the cut-off current is 1/10 C (0.3 A) for cyclic and profile aging cells and 1/20 C (0.15 A) when recharging calendar aging cells. For the profile aging cells, the driving profile is repeated until the SoC estimated before the start of the next profile falls below 10%. The discharging power may be reduced dynamically so that the cell voltage does not fall below 2.5 V.

During a CU, the charging and discharging current rate for all cells is 1/3 C (1.0 A). The cut-off current for the capacity check is 1/20 C (0.15 A).

The cycler uses voltage limits instead of SoC limits since they can be determined more reliable and reproducible than the SoC, and aging is expected to depend on the voltage rather than the SoC. The corresponding idle voltages for the calendar aging cells (V_cal,idle_) and end-of-charge or -discharge voltages for the cyclic and profile aging cells (V_cyc,lim_) are listed in the test condition table in Fig. 2. They were defined so that the relaxed voltage of a new cell after CC-CV charging or discharging with the cut-off currents indicated in the test condition table in Fig. 2 matches the corresponding OCV of a new cell at the target SoC closely.

### Test procedure

**Fig. 3:**
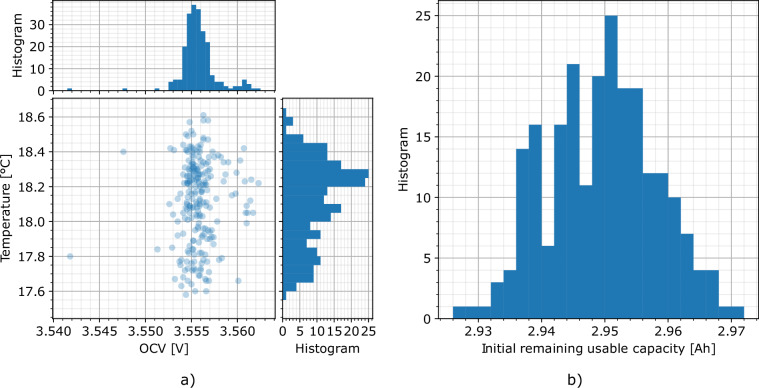
(**a**) OCV distribution over temperature during the OCV measurement of the pristine *LG INR18650HG2* cells before the experiment (including 228 cells used in the experiment and one extra cell for test purposes), (**b**) initial remaining usable capacity of the 228 cells in the experiment at the first CU.

We purchased 250 of the battery cells commercially and received them on August 30, 2022. Based on the codes lasered onto the cells (“DT331K262A_”, “_” = 1–9, B, C, or D), we conclude that the production date was likely on November 26, 2020 (T = 2020, 331 = day of the year). Once the cabling of the 228 cells used in the experiment was prepared, we measured their OCV at an ambient, steady-state temperature of 17.6–18.6°C on September 19, 2022. The mean OCV was 3.5558 V (+0.0065 V/−0.0140 V) with a standard deviation of 0.0020 V (see Fig. 3). Using the SoC-OCV dependency in Table S1 in the *Supplementary Information* document, this translates to an SoC of about 26.8% (+0.6%/ − 1.15%).

**Fig. 4:**
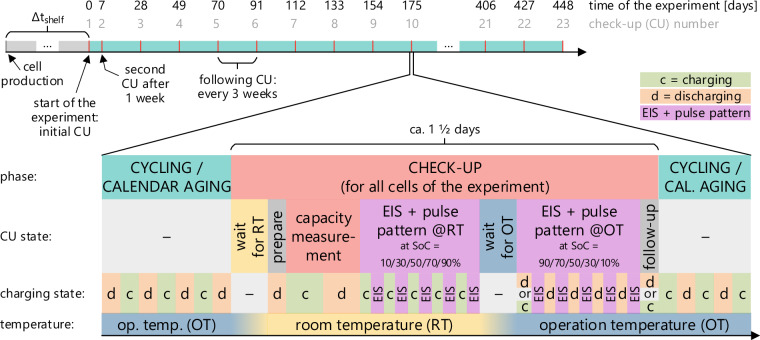
Test procedure for all cells in the experiment including detailed composition of a check-up.

The experiment began with the first CU on October 12, 2022. The second CU followed a week later. Every other CU is conducted at a three-week interval, as shown in Fig. 4. The dataset^1^ includes measurements for the first 449 days of the experiment.

The cyclic and profile aging cells are charged and discharged continuously in regular operation. Between all charging and discharge processes — in regular operation and during check-ups — rest periods of five minutes are inserted. The calendar aging cells remain at their idle voltage and are only recharged if their OCV deviates by more than 5 mV from the target voltage. A comparison of unused cells not connected to the test bench and unused cells that are connected to the cycler shows that a gradual, unwanted discharge of the cells is caused almost exclusively by the leakage current of the cycler’s measuring circuit and not by the self-discharge of the cell (which is <1%/year).

The CUs offer the opportunity to compare the estimated remaining usable capacity and the internal impedance of all cells under comparable conditions. Before the CU starts, all cells complete their ongoing cycling activity and stop after they have been discharged to their lower SoC limit. The temperature of all cells is changed from operating temperature (OT, 0–40°C) to room temperature (RT, 25°C). The temperature change takes approximately 1 1/2 hours. Once the temperature of the cells is stable, the cells are discharged to 2.6 V using their operational current settings and then discharged to 2.5 V using the check-up current rate (1/3 C) with the check-up cut-off current (1/20 C). Next, the remaining capacity of the cell is determined by a complete CC-CV charge-discharge cycle from 2.5–4.2 V with 1/3 C and a cut-off current of 1/20 C. Afterward, while remaining at room temperature, the cells are charged to an SoC of 10%, at which an EIS is conducted with an amplitude of ±1/6 C (±0.5 A) in a frequency range of 50 mHz to 14.7 kHz. The cell is charged or discharged to the nominal SoC of the EIS using the voltages shown in Table S1 in the *Supplementary Information* document. If the idle voltage is larger than V_chg_ before the EIS measurement, the cell is discharged to V_dischg_. If, instead, it is smaller than V_dischg_, it is charged to V_chg_. If the voltage lies in between, the cell is charged or discharged to V_avg_. This assures that the relaxed cell voltage is as close as possible to V_avg_ of the desired SoC.

After the EIS, a current pulse pattern is applied to the cells, and the voltage response is captured. The pulse pattern contains rectangular current signals in the range of ±1/3 C (±1.0 A). The EIS and pulse pattern measurement are repeated at 30, 50, 70, and 90%. Once all cells complete the tests, the temperature of all cells is changed back to their respective operating temperature. After the temperature reaches a steady state, the EIS and pulse pattern measurements are repeated at OT and the same SoC points in descending order. Cells that finished the last measurement will end the CU procedure by charging or discharging to the lower SoC limit of their operating condition (or idling SoC for calendar aging cells). The cycling and profile aging cells will then continue regular cycling, beginning with a charge procedure.

The check-up sequence is also shown in Figure S1 in the *Supplementary Information* document, in which selected measurement signals from the log dataset^1^ are annotated with the corresponding states of the scheduler that coordinates the CU.

The cells are operated until their estimated usable capacity falls below 40% of the nominal capacity during regular cycling or below 50% of the nominal capacity in the capacity check of a CU. This relatively low threshold for the capacity-based end-of-life (EOL) criterion was used to overcome the limitations of existing datasets and models, which often only consider aging up to 70% of the nominal capacity^6^^, p. 701^. Considering the development of capacity and impedance after this threshold allows, for instance, estimating the remaining value of an EV battery or understanding how it can be optimally operated in a Second Life application.

The estimated usable capacity is calculated after each sufficiently long charging or discharging process by dividing the actual charge difference ΔQ of this half-cycle through the SoC difference ΔSoC (see Equation (1)). The start SoC is determined just before the charging or discharging process starts. The end SoC is estimated after an idle time of five minutes after charging or discharging is finished. This also allows estimating the remaining capacity outside the CUs, e.g., for cells only operated in a 10–90% window.

1 $${C}_{estimated,remaining,usable}=\frac{\Delta {Q}_{half-cycle}}{\Delta So{C}_{half-cycle}}=\frac{\Delta Q({t}_{cycle,end})}{SoC({t}_{cycle,end}+300\,s)-SoC({t}_{cycle,start})}$$

### Experimental setup

**Fig. 5:**
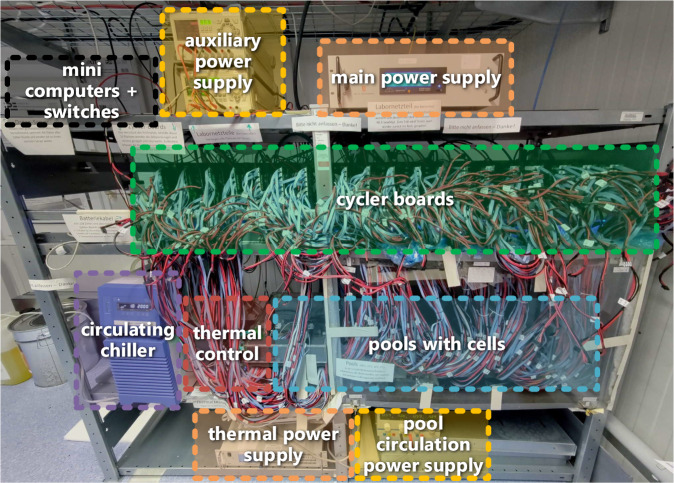
Overview of the battery aging test bench.

Cycling hundreds of cells over a period of months to years and measuring the capacity and impedance change is a costly process. Since we did not have access to a testing facility covering all the required cycling channels for the desired duration, we developed a custom, cost-efficient yet highly accurate and flexible test bench to conduct the experiment (see Fig. 5).

The cells are cycled and measured by 19 custom battery cycling and measurement acquisition boards (compare Figure S2 in the *Supplementary Information* document), each controlling 12 cells. An early prototype of this device was presented in more detail in a previous publication^13^. The basic operating principles and specifications of the cycling and the measurement hardware remained similar.

**Fig. 6:**
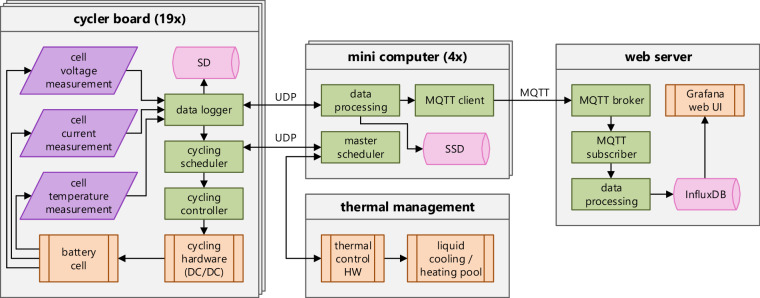
Simplified structure of the cycling test bench, including the data flow — adapted from^13^.

As shown in Fig. 6, the cycler board schedules the charging and discharging or dynamic operation modes of each cell. The cycling controller controls the respective DC/DC converter on the board to apply the desired current/power and voltage to each cell. In steady state, we measured an unfiltered voltage and current ripple at the cell of less than 5 mV and 70 mA, respectively (including measurement noise of the oscilloscope). The 20 MHz bandwidth-limited ripples are below 1 mV and 15 mA. The voltage and current signals captured by the cycler are further filtered in the analog measurement circuit. The typical measurement accuracy is within ±0.05% for the cell voltage and ±5 mA for the current, corresponding to 0.1% at the maximum charging current^13^. A zero-current adjustment for the current measurements of the analog-to-digital converter (ADC) is conducted at the startup of the controller. Even after an uptime of over a year, the measured cell current offset for a resting cell (DC/DC converter turned off) was less than 2 mA, and the logged current fluctuated by less than ±3 mA.

The cell’s voltage, current, and temperature are measured and logged with a temporal resolution of two seconds, along with states and derived variables such as the charge (ΔQ in Ah) and energy (ΔE in Wh) and the estimated SoC. The logging and publication of raw data with this relatively high resolution was used to overcome an additional limitation of existing datasets, which often only include result data or data at a significantly lower resolution. In contrast, our dataset^1^ allows for deriving battery degradation models with high temporal resolution (compare^6^^, p. 701^). The data is stored in a local SD card and forwarded to *Raspberry Pi* computers, where it is also stored and forwarded to a web server. On the server, the data is archived in an *InfluxDB* database. It can be visualized live with a *Grafana* web interface (compare Figure S1 in the *Supplementary Information* document). The master scheduler on one of the *Raspberry Pi* computers coordinates the CUs with all 19 cycler boards and the thermal management system.

**Fig. 7:**
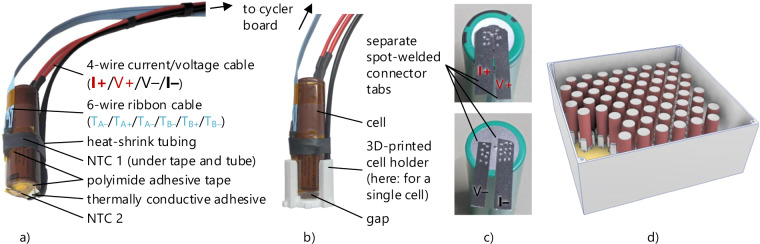
(**a**) Assembled battery cell with two NTC temperature sensors, (**b**) mounted into a cell holder, (**c**) spot-welded four-wire measurement connector tabs, (**d**) illustration of the cell arrangement in a pool in the combined cell holder.

Each cell is connected to the cycler boards using separate wires and current collector tabs for the current flow and voltage measurement, as shown in Fig. 7. This assures that the terminal voltage of the cell itself and no voltage drops across the cables or current collectors are measured. Two negative temperature coefficient (NTC) temperature sensors are mounted onto the cell’s surface using a thermally conductive adhesive. One NTC is placed at the negative pole of the cell. A second one is fixed in the center of the side of the cell. Both temperatures are measured, but only the average value is logged. If the temperatures deviate by more than 3 K, the hotter of the two temperatures is used instead of the average temperature. The cells are inserted into a 3D-printed structure covering only a small part of the cell’s body.

**Fig. 8:**
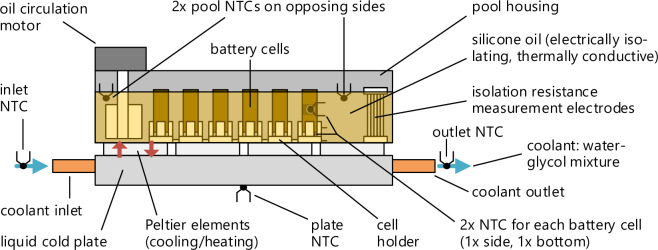
Side view of one of the pools containing the battery cells.

The cells are placed inside metal containers filled with a thermally conductive but electrically isolating silicone oil (*Julabo Thermal H20S*). A side view of one of these pools is shown in Fig. 8.

**Fig. 9:**
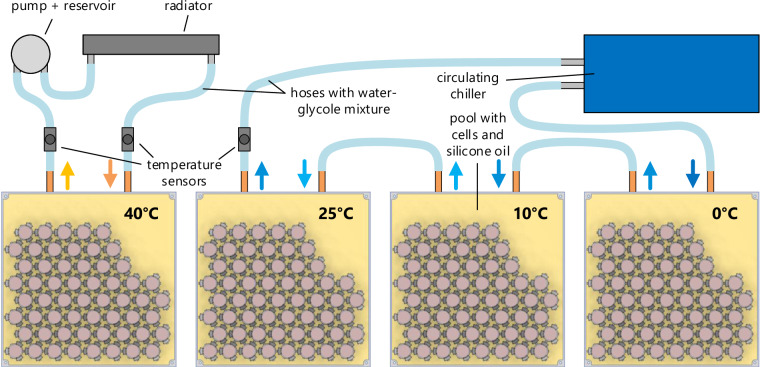
Arrangement of the pools containing the battery cells in the cooling/heating circuits.

The oil temperature, measured using two NTC sensors on opposite sides of the pool, is regulated through a thermal management system. The oil is swirled in the pool to obtain a homogeneous temperature. Cells with high current rates are placed close to the circulating blade for improved temperature stability. A water-glycol mixture flows through a cold plate placed under the pools. As illustrated in Fig. 9, the cold plates of the three cold pools (0, 10, 25°C) are connected to a circulating chiller that cools the plates. The plate of the hot pool (40°C) is connected to a radiator so the pool can exchange heat energy with the environment. Peltier elements are connected between the plates and the pools with thermal grease. They are controlled by the thermal management board and can either heat or cool the pools to reach the desired temperature.

The measured pool temperature is within ±0.5 K of the set temperature in regular operation. However, the surface temperature of individual cells can deviate by ±2 K in mild conditions, primarily due to the self-heating of the cells. The surface temperature of severely aged, fast-charging, cold cells can even reach temperature increase peaks of up to 11 K despite the liquid cooling. These individual fluctuating temperature deviations caused by the power dissipation or side reactions in the cell must be considered when the data is used to model battery aging accurately. This highlights the importance of the published raw log dataset^1^, which captures all measurements with a two-second resolution.

### Data post-processing

The published data^1^, described in the Data Records section, is based on the data stored locally on the SD cards on the cycler and thermal management boards (compare Fig. 6) since this dataset is most complete as well as easy and fast to process. The SD card data was backed up manually during the transition from room to operating temperature in check-up 23 on January 4, 2024, while the cycler boards were inactive. The raw, densely packed data on the SD cards is unpacked and decoded by Python scripts. For example, bit fields are converted to integers, and physical values stored as integers are converted to floats in the desired (mainly SI) units.

The data stored on the SD cards has no timestamp but includes an uptime counter value of the main processor, which is incremented every 0.01048576 s. A Unix timestamp is assigned when the data arrives in the mini computers and is stored along with the data on their SSDs and in the InfluxDB database on the server. During post-processing of the data, a Python script compares characteristic values of the uptime counter between the SD and InfluxDB data to identify matching data uniquely and then assigns the according timestamps of the InfluxDB data to all data rows. Timestamps during Ethernet faults or in the first seconds after the cycler reboots are estimated based on the difference in uptime values, as indicated in the timestamp_origin column.

The following data corrections were conducted:

- Due to a software bug, all PULSE data measurements stored on the SD card were incomplete. Therefore, the script extracts the unaffected PULSE data from the InfluxDB database instead of using the SD card record.
- Implausible values of the coulomb or energy efficiency (<10% or >200%) and the estimated remaining capacity (<0 Ah) are replaced with Not a Number (NaN) in the end-of-charge (EOC) dataset. They can occur if short transitional charging or discharging processes were mistakenly used to estimate these variables (e.g., during the preparation or follow-up of a CU).
- The internal flash of the main processor of the cycler board stores and updates variables that need to be recovered after reboots, such as the charge and energy that each cell processed in the current operation (ΔQ, ΔE) and since the beginning of life (Q/E_total_), the estimated remaining usable capacity (C_remaining_) and internal impedances measured in the last check-up, the initial reference impedance (Z_ref,0_), and the number of cycles and check-ups (N_cyc/CU_). The flash is updated every five minutes and before a soft reboot is requested by the user or the software to avoid data loss. If there is a sudden power loss (e.g., during electricity outages or because of a reboot initiated by the auxiliary processor due to a communication issue), the cycler board may reboot itself and continue operation with the latest variables stored in the flash. This causes steps in the ΔQ/E and Q/E_total_ data in the cell LOG and EOC dataset. They are eliminated in the post-processing script by shifting the values after reboot to the value just before reboot to repair the data as if there had been no reboot.
  Manual power cycling during a flash update operation of the cycler board caused data loss in three instances. It occurred on slave 6 on October 19, 2022, around 17:00:00 UTC (included in the published dataset), as well as on slave 14 on January 4, 2024, 15:03:13 UTC, and slave 16 on January 4, 2024, 15:28:33 UTC (not included in version 1 of the published data). After the reboot, the cycler slave board continues operation according to the schedule of the master scheduler (i.e., a check-up is continued). However, the variables mentioned earlier are reset to zero or NaN. While the SoC and OCV estimation of the battery management system (BMS) of the cycler is impaired between the data loss and the next check-up, the variables stored in the EOC dataset (number of cycles, number of check-ups, total processed charging and discharging charge and energy) and the EIS dataset (impedance-based State of Health (SoH)) were repaired by the post-processing script without impairments using the data previously stored on the SD cards.

Several plausibility checks in the post-processing script were introduced to notify the user about potential issues with the data, as described in the Technical Validation section. They helped to detect and fix the issues mentioned before. In the final run, the script executed without error messages, i.e., all plausibility checks have been passed.

**Fig. 10:**
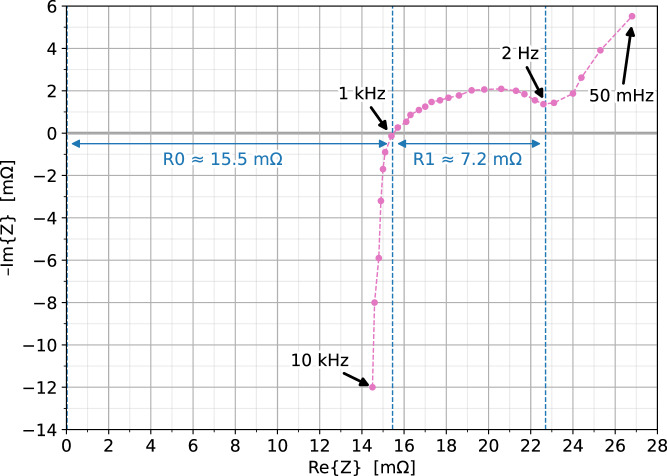
Example of an EIS measurement at 25°C, 50% SoC with the two characteristic resistances R_0_ and R_1_ of the LOG_AGE dataset (P047-2, S14:C04, Feb 2, 2023 at 00:25:41 UTC). As usual for EIS diagrams in the literature on battery cells, the imaginary axis is mirrored (−Im{Z}), which means that positive imaginary parts of Z (inductive behavior) are located on the lower half of the diagram.
- All NaN values in the OCV estimation (e.g., just after a cycler board reboot) are replaced with the measured terminal voltage in the LOG and LOG_AGE datasets.

The datasets were extended or altered as described in the following:

- The cell LOG and EOC datasets were extended by the ΔQ_chg_, ΔQ_dischg_, ΔE_chg_, and ΔE_dischg_ columns, which only consider charged or discharged charge and energy during the current run. Further, the ΔdQ_chg_, ΔdQ_dischg_, ΔdE_chg_, and ΔdE_dischg_ columns are introduced in the cell LOG dataset to provide the charge or energy difference since the last data point. The data was calculated using the ΔQ and ΔE columns of the cell LOG file. For a highly dynamic operation (e.g., because of EIS measurements or driving profiles), these values may slightly underestimate the actual charge or discharge charge (Ah) or energy (Wh) since they are based on data with only a two-second resolution. In contrast, the internal ΔQ variables of the BMS are updated with 1 kHz in the cycler.
- Similarly, Q/E_total,CU-RT/CU-OT/others-RT/others-OT/sum,chg/dischg_ (20 columns) were added to the cell LOG and EOC datasets to easily contribute the total charge and energy to specific operating conditions (e.g., check-up and other transitional charging and discharging processes at room or operating temperature), which might assist battery degradation modeling.
- There are gaps in the dataset ranging from several seconds to about one day, caused mainly by Ethernet issues (particularly in the first two months of the experiment) but also because of local power outages. The cycler is configured only to cycle the cells if both the SD card and internet logging work. The cells rest during data gaps, and the pool temperatures are maintained. However, the last valid data point often contains a row where the cell is still cycled, i.e., the current and power are not zero. Simple interpolation using standard methods (e.g., with the pandas Python library) would not accurately reflect the behavior in this case. In order to prevent this, a new artificial data point is inserted in the cell LOG dataset just after the last valid and just before the first valid measurement during a data gap. In these new data points, the cell current and power are set to zero, and the scheduler state is set to the pause state. The new data points are inserted for every gap lasting more than 60 seconds (or 20 seconds if the scheduler state at the first valid point is undefined, indicating a reboot).
- The published LOG data only includes measurements of the cell while it is still active in the experiment, which significantly reduces the size of the published data. If the cell was permanently disabled due to an EOL or another permanent fault condition, the raw log data is cut three days after the cell was active for the last time.
- Since the EIS measurement is based on a relatively low-cost circuit, the results are less accurate than with significantly more expensive commercial equipment. The measurement accuracy of EIS results captured by the cycler was compared to measurements from a *BioLogic VSP* device. The average absolute error between the two EIS measurements was 1.54% for the amplitude and 1.03° for the phase, with individual outliers of up to 13.17% for the amplitude and 4.3° for the phase error^13^.
  Nevertheless, the EIS dataset clearly shows the development of the various components of the frequency-dependent impedance at different temperatures, SoCs, and over time under different aging conditions. The published dataset includes additional columns with compensated and filtered impedance values to improve the data quality. The compensation was necessary because the measurement method changes with decreasing frequency. The measurement method used at frequencies above 100 Hz is hardware-based and experiences a phase shift, which increasingly becomes apparent from 500 to 100 Hz. This phase shift is minimized through the compensation. The method used at 100 Hz and below is software-based and more robust for medium frequencies but also experiences inaccuracies, particularly at 100 Hz and frequencies below 0.5 Hz if the impedance is small. The frequency range, in which the data is most accurate is between 0.5 Hz and 5 kHz, excluding data points at 100 Hz and 208.3 Hz. Implausible values, in which the amplitude or phase difference to neighboring points is unusually high, are set to NaN. The point at 14.7 kHz is always set to NaN since it is visibly implausible most of the time, but this could not be detected well through the processing script. The filtering conditions were intentionally chosen to be relatively relaxed to avoid removing unnecessarily many data points. This means that the published EIS measurements still contain individual data points with visibly higher inaccuracy, as can be seen in the published EIS plots included in the dataset. Please make sure the data quality of the EIS measurement is sufficient for your application and further filter implausible values according to your needs.
- A new LOG_AGE dataset is introduced, which only includes the most important columns of the cell LOG at a reduced time resolution (30 s for cyclic and calendar aging cells, 2 s for profile aging cells). Unlike the timestamp column of all other datasets, the timestamp is not a Unix timestamp but the time since the start of the experiment in seconds (see Data Records section). Data gaps are interpolated, and the data is averaged to obtain data rows in uniformly spaced time steps. Moreover, three new columns are inserted, which include measurements of the CUs: The estimated remaining capacity C_remaining_ and the characteristic resistances R_0_ and R_1_. C_remaining_ (determined in the discharge capacity measurement of the CU) is simply copied from the EOC dataset and inserted into the LOG_AGE dataset at the corresponding time. R_0_ and R_1_ are derived using all EIS measurement results from a CU. They are calculated individually for each EIS measurement, averaged for all valid measurements of the CU, and then added in the LOG_AGE data at the timestamp following the timestamp of the last EIS measurement of this CU. As demonstrated in Fig. 10, R_0_ is defined as the resistance from the origin to the intersection of the impedance curve with the real axis. R_1_ is the real part of the section between this intersection and the EIS measurement point around 2 Hz, which has the smallest absolute value of the phase.

## Data Records

The data^1^ is freely accessible under the CC BY 4.0 license on *RADAR4KIT* (https://radar.kit.edu/radar/en/), a research data repository of the *Karlsruhe Institute of Technology (KIT)*.

The formal start of the experiment was on October 12, 2022, at 16:45:00 UTC (Unix timestamp: 1665593100). Version 1 of the published dataset covers a test duration of 449 days, ranging from October 12, 2022, at 18:20:00 UTC (Unix timestamp: 1665598800) to approximately January 4, 2024, 13:42 UTC (Unix timestamp: 1704375720).

The following list gives a brief overview of the different datasets^1^. Detailed information about every column, the data type, precision, unit, and plausible minimum and maximum values are provided in the “Data Structure” Excel spreadsheet in the published dataset^1^.

- **Configuration files (CFG):** Formal definition of the operating conditions and settings used for the cells (compare Fig. 2), pools, and slaves (cycler and thermal management boards). The configuration was stored on the SD cards before they were inserted into the slave boards. The boards read the configuration after booting and behave accordingly.
  - *228* + *4* + *1* + *19 comma-separated values (CSV) files, total size:* <*1 MB, compressed:* <*1 MB*.
  - **Cell configuration:** 228 files, e.g., *cell_cfg_P032_2_S07_C00.csv* for parameter ID 32, nr. 2, slave 7, channel 0.
  - Columns (28): *slave and cell ID; parameter ID and number; aging type, temperature, SoC, charging and discharging rate; maximum and minimum limits of charging and discharging voltages and (cut-off) currents during cycling, check-ups, and pulse patterns;* …
  - **Pool configuration:** 4 files, e.g., *pool_cfg_T00_P1.csv* for pool 1 of the thermal management board (slave 0).
  - Columns (7): *slave and pool ID; operation and check-up temperature;* …
  - **Slave thermal management configuration: ***slave_cfg_T00.csv* for the only thermal management board in the system (slave 0).
  - Columns (6): *slave ID and type, MAC and IP addresses, SD block location at which the configuration was stored*
  - **Slave cycler configuration:** 19 files, e.g., *slave_cfg_S18.csv* for cycler board slave 18.
  - Columns (17): *slave ID and type, MAC and IP addresses, SD block location at which the configuration was stored, number and SoC of EIS points*
- **Post-processed end of charge data (EOC):** Collected after each run (i.e., a charge, discharge, or driving profile operation). Contains information about the operating condition and statistics of the last run.
  - *228 CSV files, total size: 397 MB, compressed: 77.2 MB*.
  - For example, *cell_eocv2_P076_3_S17_C02.csv* for parameter ID 76, nr. 3, slave 17, channel 2.
  - Columns (57): *timestamp and origin; cycling and charging condition (regular operation, CU, other; charging, discharging); aging type, temperature, SoC, charging and discharging rate, profile; maximum charging or minimum discharging voltage and (cut-off) current; estimated remaining capacity and capacity-based SoH*; *ΔQ*/*E*_(*chg*/*dischg*)_* of last run and total processed charge or energy (for each operating condition) Q*/*E*_*total*,*CU*−*RT*/*CU*−*OT*/*others*−*RT*/*others*−*OT*/*sum*,*chg*/*dischg*_; *coulomb and energy efficiency; OCV, SoC, and temperature at the start and after the end of the run, cycling/run duration; number of cycles and CUs*; …
- **Post-processed electrochemical impedance spectroscopy measurements (EIS):** Frequency-dependent impedance, collected at each CU for room (RT) and operating temperature (OT) at five different SoCs (10, 30, 50, 70, 90%), in the range of 50 mHz to 14.7 kHz.
  - *228 CSV files, total size: 139 MB, compressed: 9.4 MB*.
  - For example, *cell_eisv2_P047_2_S14_C04.csv* for parameter ID 47, nr. 2, slave 14, channel 4.
  - Columns (22): *timestamp and origin; charging and temperature condition; nominal SoC; valid flag; current and initial reference impedance* (*Z*_*ref*_, *Z*_*ref*,0_); *impedance-based SoH; average estimated OCV and temperature during the measurement; duration of the EIS; frequency and impedance amplitude and phase for each measurement point (raw and compensated); real and imaginary part of the impedance (compensated)*; …
- **Post-processed pulse pattern measurements (PULSE):** Data of the pulse current pattern applied after completion of the EIS at each CU for room (RT) and operating temperature (OT) at five different SoCs (10, 30, 50, 70, 90%).
  - *228 CSV files, total size: 208 MB, compressed: 19.8 MB*.
  - For example, *cell_plsv2_P074_3_S19_C03.csv* for parameter ID 74, nr. 3, slave 19, channel 3.
  - Columns (19): *timestamp and origin; charging and temperature condition; nominal SoC; aging type, temperature, SoC, charging and discharging rate, profile; average temperature during the measurement; 10 ms and 1 s pulse resistance; voltage and current for each measurement point;* …
- **Post-processed extended cell log (LOG):** Contains post-processed log data for every cell collected with a two-second resolution during the experiment and further extended by the columns described in the Data post-processing chapter.
  - *228 CSV files, total size: 878 GB, compressed: 55.7 GB*.
  - For example, *cell_logext_P007_1_S06_C11.csv* for parameter ID 7, nr. 1, slave 6, channel 11.
  - Columns (59): *timestamp and origin; raw cell voltage, current, power, and temperature*; Δ*Q*/*E*_(*chg*/*dischg*)_* of current run; incremental *Δ*dQ*/*E*_*chg*/*dischg*_* since the last LOG entry; total processed charge or energy (for each operating condition) Q*/*E*_*total*,*CU*−*RT*/*CU*−*OT*/*others*−*RT*/*others*−*OT*/*sum*,*chg*/*dischg*_; *rough estimation of the OCV and SoC; measurement, BMS, voltage/current/temperature measurement state; scheduler state (raw and decoded into individual sub-states); overcharge, undercharge, and health flags indicating the capacity- or impedance-based EOL*; …
- **Compact cell log file with aging variables (LOG_AGE):** Compressed version of the LOG dataset, containing only the most essential columns at a reduced, uniform time-resolution (interpolated and averaged data without gaps), extended by capacity and impedance aging data.
  - *228 CSV files, total size: 48.9 GB, compressed: 5.1 GB*.
  - For example, *cell_log_age_30s_P035_2_S09_C04.csv* for parameter ID 35, nr. 2, slave 9, channel 4 (cyclic aging, 30 second resolution) or *cell_log_age_2s_P068_2_S07_C03* for parameter ID 68, nr. 2, slave 7, channel 3 (profile aging, 2 second resolution).
  - Columns (11): *timestamp; raw cell voltage, current, and temperature; rough estimation of the OCV and SoC*; Δ*Q* *of current run; total equivalent full cycles (EFC) of this cell in the experiment; estimated remaining capacity of the cell; estimated characteristic resistances R*_0/1_* of the cell* (*compare* Fig. 10)
- **Raw pool log (POOL_LOG):** Raw, unprocessed log data for every pool collected with a two-second resolution during the experiment.
  - *4 CSV files, total size: 12.0 GB, compressed: 674 MB*.
  - For example, *pool_log_T00_P3.csv* for pool 3 (40°C) of thermal management slave 0.
  - Columns (46): *timestamp and origin; scheduler state (raw and decoded into individual sub-states); pool set and measured temperature; cold plate temperature; Peltier currents and states; RT/OT, stable, and timeout flags;* …
- **Raw slave log (SLAVE_LOG):** Raw, unprocessed log data for every slave (cycler and thermal management) collected with two-seconds resolution during the experiment.
  - *1 thermal management* + *19 cycler board CSV files, total size: 34.4 GB, compressed: 2.83 GB*.
    - **Slave thermal management log:** *slave_log_T00.csv*.
    - Columns (47): *timestamp and origin; last completed master scheduler state; slave scheduler and controller state; main power supply voltage, current, and power; auxiliary power supply voltage; main and auxiliary voltage (and current) states; emergency stop, DC link, and log states; SD card filling level; processor uptime; air valve, board and radiator fan, pump enable flag; circulating chiller set and measurement temperature and speed; pool isolation resistance measurement and state; coolant temperatures and states; ambient and “cold box” temperature, dew point, pressure, relative and absolute humidity;* …
    - **Slave cycler log:** 19 files, e.g., *slave_log_S01.csv* for cycler board slave 1.
    - Columns (21): *timestamp and origin; last completed master scheduler state; slave scheduler and controller state; main power supply voltage, current, and power; auxiliary power supply voltage; main and auxiliary voltage (and current) states; emergency stop, DC link, and log states; SD card filling level; processor uptime;* …
- Additional files:
  - **Cycling Experiment Cell Overview**: 1 Excel spreadsheet
  - Overview of the aging conditions, assignment of parameter set ID (Pxxx) + number (-x) to cycler slave boards (Sxx) and channels (:Cxx), position of the cells in the pools.
  - **WLTP cycle power profile:** 1 Excel spreadsheet
  - Power profiles derived from the WLTP that were used in the experiment.
  - **Data Structure**: 1 Excel spreadsheet
  - Formal definition of the structure of all datasets, e.g., names, data types, plausible limits, and description of each data column, as well as description of all state variables. The “help” tab of the spreadsheet provides an overview and brief description of the published data records. Similar data records are combined into one table, e.g., the cell, pool, slave cycler, and slave thermal management configuration are described in the “CFG” tab of the spreadsheet, and the slave cycler and slave thermal management log are shown in the “SLAVE_LOG” tab.
  - **Battery Aging Data Plausibility Check:** 1 Excel spreadsheet
  - Python-generated summary table showing the minimum, maximum, and number of NaN values for each dataset column, as well as the number of values outside of the plausibility limits defined in the Data Structure spreadsheet.
  - **Result plot examples:** interactive .html files visualizing various EIS, EOC, and PULSE result data columns in different configurations (e.g., grouped by temperature or SoC, visualization of the development of the impedance over time, …) — the plots (for example, the figures shown in the section “Brief evaluation of the measurement results”) were generated by the *plot_result_data_comparison.py* Python script (see *Code availability* section).
  - *864 HTML files, total size: 8.44 GB, compressed: 148 MB*

Most of the data records are published as compressed *.zip* files. However, large files are compressed in the *.7z* format since the compression is faster and the compressed files are significantly smaller. This *.7z* archive can be unpacked using the free and open-source software 7-Zip (available for Windows, Linux, and macOS on https://www.7-zip.org) or libraries such as the Python py7zr library (https://py7zr.readthedocs.io/).

## Technical Validation

### Data quality and inspection

Although we did not use commercial battery cycler and measurement systems, our custom cycling system was tested and optimized at length before starting the experiment. Nevertheless, several minor problems emerged during the test period, such as sporadic Ethernet failures in the first two months of the dataset, which caused data gaps during which the cycler was automatically stopped to minimize the loss of relevant measurements. In order to ensure the high quality of this large dataset^1^, we continuously monitored samples of the data in Grafana, kept optimizing the system to minimize interruptions, and used Python scripts to analyze the final data for anomalies automatically. The impacts of the initial software bugs, such as partly incomplete data packets on the SD card, were fixed in the published data as described in the Data post-processing chapter so that the data quality is not affected.

Among others, the following checks were implemented on the battery cycler boards to ensure high data quality and safety:

- All relevant measurements, such as the cell voltage, current, and temperature, are constantly monitored in a control loop executed with 10 kHz for safety and control purposes. The cell voltage is measured with a resolution of 69 *μ*V and an accuracy of less than 0.05% in typical operating conditions. The current is captured with a resolution of 343 *μ*A and an accuracy of typically less than 0.1%^13^. In the relevant operating range, the cell’s temperature is measured with a resolution of less than 0.05 K, and an estimated accuracy of 1 K. The tolerance of the crystals used to derive the processors’ clock signals (relevant for the processor uptime counter and the EIS frequency) is less than ±25 ppm, including frequency tolerance, frequency-temperature variation, and aging effects.
  Derived values such as the OCV, SoC, ΔQ, and ΔE are monitored with a frequency of 1 kHz by the BMS (implemented on another core of the cycling processor) for safety purposes. If a safety-relevant value is NaN or surpasses critical upper or lower limits, or surpasses warning limits for a specified time, the cell will be temporarily disabled, i.e., left floating. After the fault is cleared and a resting period has passed, the cycler tries to re-enable the cell. After multiple faults during a defined time frame, the cell will be permanently disabled. If safety-critical fault states, such as an over-charge, occur, the cell will be immediately disabled permanently.
- The combined current and voltage controller operates in a closed loop, assuring that deviations are corrected within a time frame of typically less than 5 ms for the cell current and less than 30 ms for the voltage. If the controller cannot accurately set the desired current and the cell is not close to the end-of-charge or -discharge voltage, a controller error will be raised, which disables the cell channel. For example, this can occur if the cell fuse on the cycler board is blown.
- Every Ethernet data transfer between the slaves and the mini computers is acknowledged by the receiver of the data, and the successful storage of data on the SD card is also monitored. Failure to send or write the data will increment a fault counter. However, only one attempt is made to store LOG data (for slaves, cells, and pools) on the SD card and send it to the mini computers via Ethernet. All other data records generated by the cycler (EOC, EIS, PULSE) are sent and stored until a confirmation about the successful arrival/storage of the data is received to avoid result data loss.
- If a logging fault occurs for more than 20 seconds, the cycler stops operation so that no relevant cycling data is lost. The thermal management boards maintain their temperature when logging fails.
- As mentioned, three cells were tested per operating condition. The cells were distributed among the cycler boards to ensure that all three cells aging under a specific operating condition are connected to different cycler boards and controlled by different mini computers, so the measurement, data acquisition, and storage are independent.

In the Python scripts that decode the data, assign timestamps to every data row, and repair and analyze the data (also see Data post-processing chapter), the following checks are performed:

- For each slave board, we programmatically and visually inspected all LOG timestamps on a semi-logarithmic scale to easily detect anomalies in the time difference between two data rows. Warnings are raised if timestamps are decrementing, or the difference between consecutive timestamps is unusually small or large (the latter is the case after data gaps).
- All values of all published CSV datasets are checked for NaN values and against plausible minimum and maximum thresholds, which are defined in the “Data Structure” Excel spreadsheet provided along with the data records. The resulting “Battery Aging Data Plausibility Check” spreadsheet is also included in the published dataset. While the analysis confirms that almost all values are valid and in a plausible range, the following exceptions exist.
  NaN values can occur in the following instances:
  - in the EOC data in the *cap_aged_est_Ah* (estimated remaining usable capacity), *soh_cap* (capacity-based SoH), *coulomb_efficiency*, and *energy_efficiency* columns if the charging or discharging operation was too short,
  - in the EOC data in the *coulomb_efficiency* and *energy_efficiency* columns if the charge-discharge cycle was asymmetric (e.g., in regular operation for the profile aging cells),
  - in the EIS data in the *z_ref_init* column if the first EIS measurement of a condition, determined by the SoC (10/30/50/70/90%) and temperature (RT/RT), was invalid, i.e., the EIS measurement timed out, or the cycler was severely interrupted (which happened for several cells since the test bench was interrupted in the first EIS procedure) — in these cases, the next valid EIS measurement (second or third CU) is used as the reference for this measurement point,
  - in the EIS data in the *z_ref_now_mOhm* and *soh_imp* columns for any invalid EIS measurement,
  - in the EIS data in the *z_amp_mOhm* and *z_ph_deg* columns if individual measurement points (i.e., at a particular frequency) were not stable or could not be captured reliably enough,
  - in the EIS data in the *z_amp_comp_mOhm*, *z_ph_comp_deg*, *z_re_comp_mOhm*, and *z_im_comp_mOhm* columns if the uncompensated *z_amp_mOhm* or *z_ph_deg* values are invalid or if the measured point is implausible (see Data post-processing chapter),
  - in the LOG dataset in the *v_raw_V*, *i_raw_A*, *p_raw_W*, *t_cell_degC*, and *ocv_est_V* columns if just after boot, data is logged before valid measurements have been collected — if these NaN values shall be eliminated, use the next valid measurement point as a replacement (backward filling) or remove the row (since it is the first after boot),
  - in the LOG_AGE dataset in almost all *cap_aged_est_Ah*, *R0_mOhm*, and *R1_mOhm* columns — they are intentionally only filled with valid result data values from the EOC and EIS datasets in the LOG_AGE data row that follows the time at which the result data value was measured (if needed, these values could be spread across the whole dataset using linear interpolation or according to an aging model, depending on the application),
  - in several fields in raw, unprocessed log datasets, such as the SLAVE_LOG and POOL_LOG of the thermal management board and the SLAVE_LOG of the cycler boards (see “Data Structure” and “Battery Aging Data Plausibility Check” Excel spreadsheets for details).
  Contrary to definitions often used in the literature, the capacity-based SoH in the dataset (*soh_cap*) is 100% if *cap_aged_est_Ah* equals the nominal cell capacity and 0% if it equals the minimum capacity threshold (40% of C_nom_ in regular operation, 50% of C_nom_ in CUs). Therefore, it can also be negative. Values that are smaller or larger than the specified plausible limits occur:
  - in the EOC data in the *cyc_duration_s* (duration of a charge or discharge operation) column if the cycler was interrupted for a longer period,
  - in the LOG dataset in the *sd_block_id* (corresponding raw data position on the SD card for traceability) if synthetic data was inserted (e.g., the pause flags marking gaps, as described in the Data post-processing section), the *sd_block_id* is set to 0,
  - in the raw POOL_LOG and SLAVE_LOG (thermal management and cycler) datasets in the *timestamp*, *scheduler_state_dec*, and *scheduler_state_phase* columns during the first three hours of the dataset, since the boards were started before the formal start of the experiment, and the thermal management board initially operated in manual operating mode in this time,
  - in the raw SLAVE_LOG datasets in the *v_aux_V* column if the auxiliary supply voltage measurement is not yet initialized after boot, but the first log data row is already written, the value is 0,
  - in the raw SLAVE_LOG dataset of the thermal management board in the *pump_hot_meas* — replace values >100% (duty cycle measurement error) with NaN or use interpolation if needed,
  - in the raw SLAVE_LOG dataset of the thermal management board in all columns from the combined temperature, pressure, and humidity sensor (*t_box_cold_degC* to *abs_hum_ambient_g_m3*, 10 columns), since the measurement and the I2C communication to the sensor using a relatively long cable is prone to noise — sudden changes of these measured and derived physical values are unlikely, so values that significantly differ from neighboring values or are otherwise implausible can be replaced with NaN or interpolated values if needed.

Besides the data gaps, there was an issue with the temperature control between November 27, 2022, 10:40 PM UTC and November 29, 2022, 16:50 UTC. The air drying mechanism was impaired, which caused the dew point in the “cold box”, where the three colder pools are placed, to rise to approximately 8°C. The thermal management stopped cooling (and heating) the pools to prevent condensed water from entering them. During this period, the temperature in the three colder pools was between 9 and 13°C (instead of 0, 10, and 25°C), and the warm pool had around 32–33°C (instead of 40°C).

In addition, one of the Peltier element circuits of the 25°C pool failed on April 23, 2023, at around 11:47 AM. As a result of the lower heating power, the average temperature of the cells in the pool decreased by about 2.5°C, and the temperature variation inside the pool increased. The Peltier element was not replaced since this would have caused a long interruption of the experiment.

### Brief evaluation of the measurement results

The published data records^1^ include more than 3 billion data rows, each containing numerous measurement, derived, and state variables. The vast majority of data record size is attributed to the LOG files. The most relevant findings are summarized by the result data records (EOC/EIS/PULSE), visualized in the “result plot examples” published among the data records using the Python scripts described in the *Code availability* section. Selected results shall be briefly summarized and put into context in the following.

**Fig. 11:**
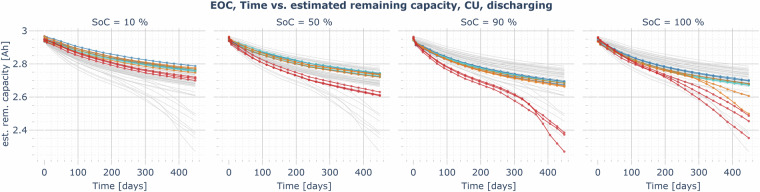
Example plot for the EOC data record: estimated remaining usable discharging capacity over time, measured in the CUs at 1/3 C and 25°C, comparison of all calendar aging cells resting at an SoC of 10, 50, 90, and 100% (blue: aging at 0°C, cyan: 10°C, orange: 25°C, red: 40°C, gray (background): overlay of the capacity trajectories of all calendar aging cells with other SoCs for comparison).

**Fig. 12:**
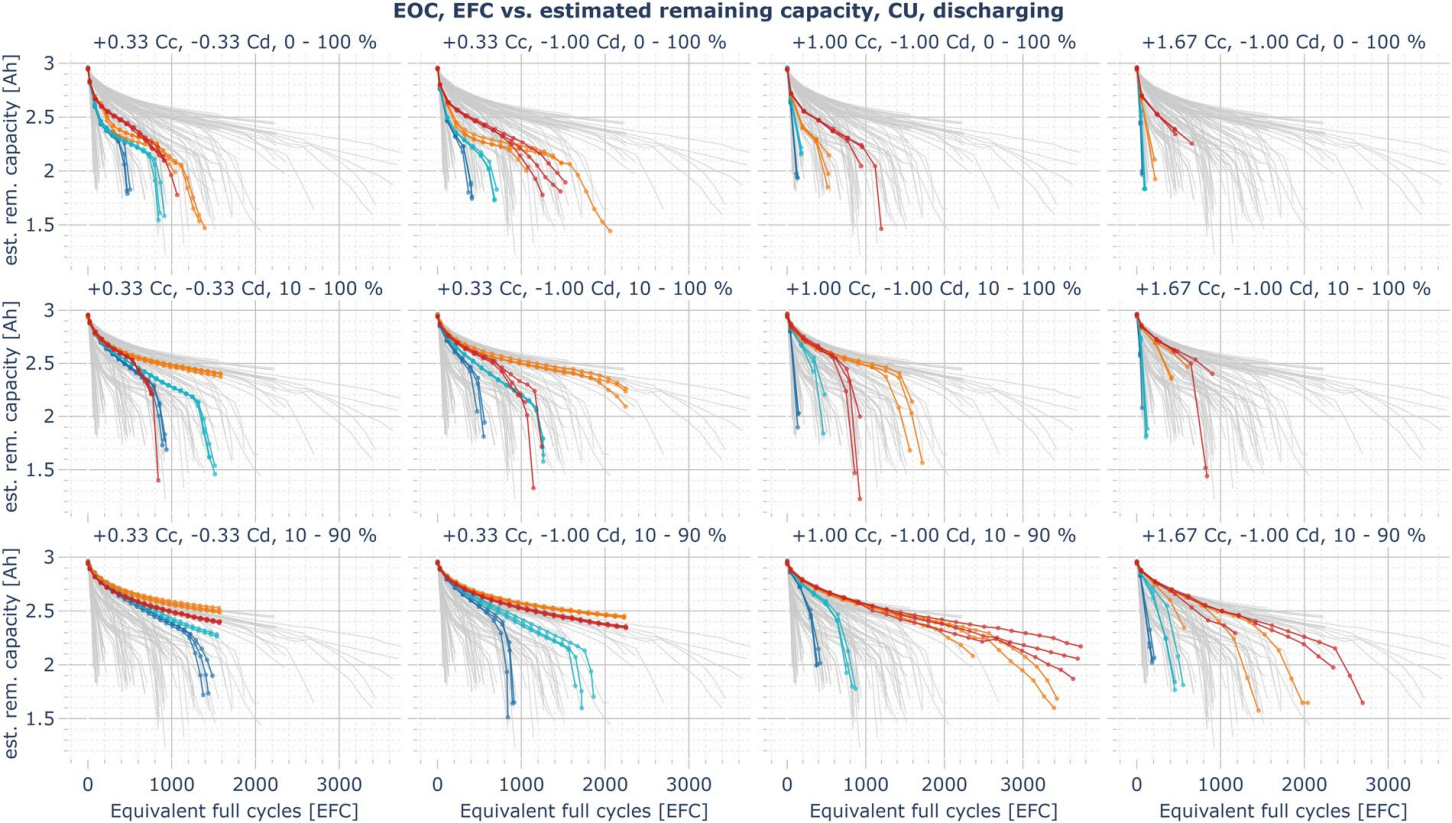
Example plot for the EOC data record: estimated remaining usable discharging capacity over the number of equivalent full cycles, measured in the CUs at 1/3 C and 25°C, comparison of all cyclic aging cells (blue: aging at 0°C, cyan: 10°C, orange: 25°C, red: 40°C, gray (background): overlay of the capacity trajectories of all cyclic aging cells (other SoC ranges, charging/discharging rates) for comparison).

**Fig. 13:**
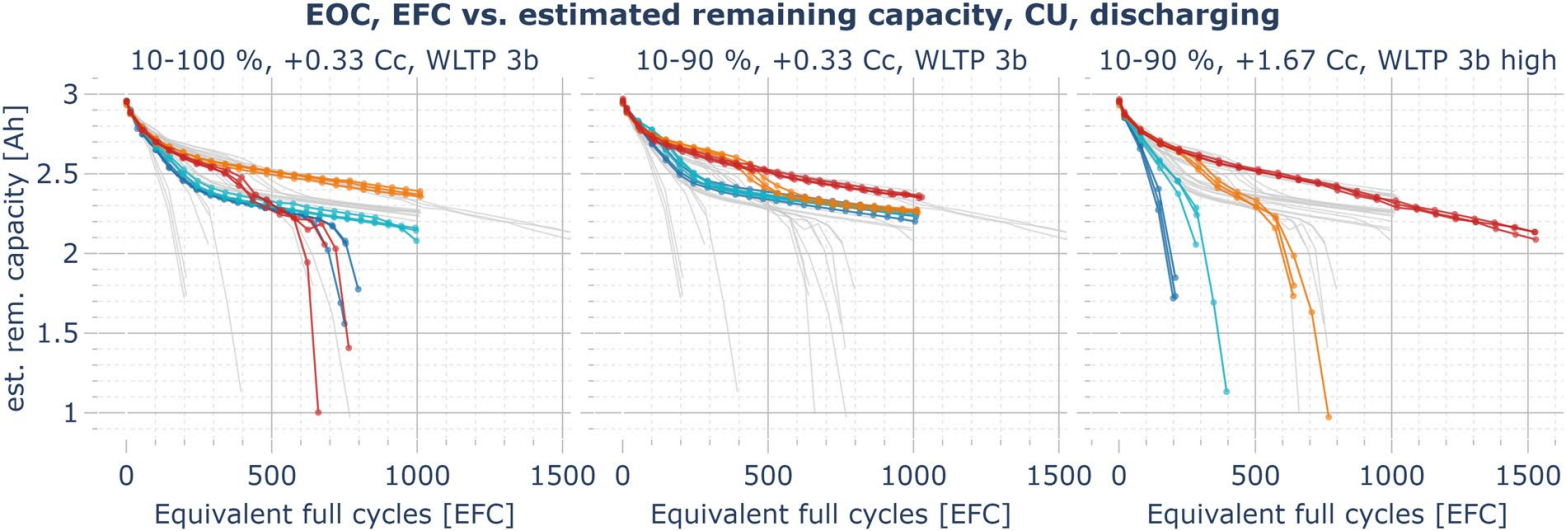
Example plot for the EOC data record: estimated remaining usable discharging capacity over number of equivalent full cycles, measured in the CUs at 1/3 C and 25°C, comparison of all profile aging cells (blue: aging at 0°C, cyan: 10°C, orange: 25°C, red: 40°C, gray (background): overlay of the capacity trajectories of all profile aging cells (other SoC ranges, charging rates and profiles) for comparison).

Figures 11 to 13 show the estimated remaining discharge capacity measured in all CUs over time or the number of EFCs. They allow comparing the capacity loss caused by different aging conditions under comparable reference conditions (see Test procedure section). In all of the figures, the aging temperature is represented by the color of the trajectories. As expected for calendar aging (Fig. 11), the capacity fade is particularly dominant at high SoCs and high temperatures. Colder cells and cells resting at lower temperatures also face considerable capacity losses, but the change rate generally relaxes over time. However, for the cells aging at 40°C and 90% as well as at 25 and 40°C at 100%, the capacity loss rate increases again after the remaining capacity decreases past 85% of the nominal capacity.

A considerable fraction of the capacity fade of all calendar aging cells might be caused by the cyclic aging caused during check-ups. After the last CU shown in the figure, the cells experienced between 43 and 63 EFCs, primarily due to the capacity checks, where the cell is charged and discharged between 0 and 100%.

A rather unexpected result is that the calendar aging at 40°C is higher at an SoC of 90% than at an SoC of 100%. However, other authors have also reported similar behavior in the past^14^^, p. 9,^ ^15^^, p. 58,^ ^16^^, p. 4^. In our case, it is still unclear which fraction of the measured capacity losses are irreversible and which part is reversible and could thus be recovered with a suitable operating strategy.

In almost all cases of the cyclic aging cells (Fig. 12), the capacity loss rate in the first 200 cycles is relatively high and then reduces. The loss rate gradually increases again when the remaining capacity is less than 70 to 85% of the nominal cell capacity. In most instances, the capacity loss rate for cyclic aging cells at cold temperatures is significantly higher. However, at slow charging rates (e.g., +1/3 C,  −1/3 C, 10–100%), where calendar losses also have a high relative impact on aging, but lithium plating is less likely to occur at cold temperatures, there is no significant difference between the aging at 0 and 40°C. In this case, cells cycling at 25°C show the lowest aging rate since both the calendar and cyclic aging losses are low. The capacity losses for cold, fast-charging cells are particularly severe, especially when charging to 100%. Lithium plating is expected to be the dominant driver of the capacity losses in these cases. In the most extreme instance (1.67 C charging rate from 0–100% at 0°C), the cells had less than 65% of the nominal capacity after just 132 EFCs. This highlights the importance of a good thermal management system and the preconditioning of an EV battery before fast charging, as well as limiting the charging rate to a value at which no plating occurs under the present conditions (e.g., by estimating the anode potential of the cell^17^). However, lithium plating can significantly drive capacity fade for severely aged cells, even at milder temperatures and lower charging rates^7^. This may explain the characteristic knee point in most of the investigated operating conditions, after which the aging is significantly accelerated. The published LOG data might help to better understand the characteristics of lithium plating and stripping processes in the cell and the dependence of the anode potential on temperature, C-rate, cell voltage, and impedance increase through aging. For example, an unusual increase in the charging current in the CV phase or the presence of an additional CC phase after a CV phase has already been reached can be seen in the LOG data (see Figure S3 in the *Supplementary Information* document). This behavior indicating lithium plating and lithium stripping is also discussed by Ringler *et al*.^17^^, p. 7^. In mild operating conditions, e.g., charging and discharging with 1/3 C from 10 to 90% at 25°C, the cell still maintains more than 80% of the nominal capacity after more than 1500 EFCs (2083–2091 EFCs at the end of the experiment in May 2024).

The profile aging cells (Fig. 13) generally show a capacity loss behavior consistent with the cycling aging cells and also confirm qualitative findings known from the literature on calendar and cyclic aging. Limiting charging to an SoC of 90% instead of 100% has a positive effect on the lifetime of cells operated at 25°C and especially at 40°C. Although not investigated with cyclic aging cells, the effect might even be higher at lower charging voltage limits, as indicated by the calendar aging results. This is particularly relevant for the operation of EVs in warmer regions and in summer. At 40°C operating temperature, the fast-charging highway driving profile faces capacity losses almost similar to the slow-charging complete driving profile. However, at temperatures of 25°C and below, the fast charging rate affects aging severely.

The fastest aging occurs at the fast-charging highway profile at 0°C pool temperature: Less than 80% of the nominal capacity remains after just 132 EFCs (roughly 32,500 km of the highway profile). In contrast, the cell operating in the 40°C pool reaches 860 EFCs before it passes the 80% threshold (roughly 220,000 km) and around 1530 EFCs until only 70% of the nominal capacity remains (roughly 390,000 km) under otherwise similar conditions.

The preliminary results of the cells aging under the full driving profile indicate that the cell aging at an SoC range of 10–100% at 25°C and 10–90% at 40°C face even fewer capacity losses.

It has to be mentioned that the selected cell model has already been on the market for a considerable time^10,18^ and was selected due to its availability and the otherwise good performance indicators relevant to EVs. Newer NMC cell models suitable for automotive applications likely reach a significantly higher lifetime^8^.

## Usage Notes

As already discussed, the published data^1^ is helpful for a wide range of applications. Although it is relatively size-inefficient, the CSV file format was chosen for the data records because it is very simple to read and process independently of the software or programming language used. Sample code for reading and displaying the dataset with Python is freely available in a Git repository (see *Code availability* section). The *read_dataframe_example.py* script is a minimal example for reading, selecting, and plotting data records. In contrast, *plot_result_data_comparison.py* is a more complex script that generates interactive plots of the result data records (EIS, EOC, and PULSE). Further, user-defined plots can be added relatively easily by adjusting the *PLOT_TASKS_LIST* structure, which defines a list of plots to be generated by the script.

In theory, the LOG data can also be plotted. However, due to the size of the data, this quickly reaches computational limits with most libraries when visualizing data from several months. Adequate filtering, data resampling to a significantly lower time resolution, or using the LOG_AGE dataset may facilitate visualization.

When using the data records for aging modeling, it is worth considering that the calendar aging cells do not only face calendar aging but also cyclic aging due to the relatively frequent CU, which may even dominate in their capacity fade. Conversely, the cyclic aging cells also face calendar aging since the cycling takes several months to years, depending on the charging and discharging rate.

Moreover, cyclic aging cells are not permanently exposed to the nominal charging and discharging current due to the CC-CV charging protocol. The CV phase accounts for a considerable part of the charging time, particularly for the fast charging rates at cold temperatures and aged cells, i.e., high internal impedances. In a severe case (P017-1: 0°C, +1.67 C/ −1.0 C, 0–100%), close to the end of the lifetime of the cell, the CC phase only lasts less than two minutes, while the CV phase until the cut-off current of 1/10 C was reached took more than one hour. While in the literature, simplified aging model functions are sometimes fit to experimental capacity fade result data using the nominal charging current as an input, it is recommended to consider the variable operating conditions for modeling. This is possible using the published log data, such as the compact and easily reusable LOG_AGE dataset. This also allows considering the effect of the individual temperature increases that the cells face depending on their operating condition and degradation state.

The SoC and OCV estimations are determined by comparatively simple algorithms and deliver inaccurate results for aged cells while charging or discharging. Therefore, they should not be used to validate SoC algorithms without prior modification. At rest, the SoC in the dataset is estimated using the low-pass filtered measured cell voltage as the estimated OCV and a lookup table (LUT) for the relationship between the OCV and the (Ah-based) SoC (using the “avg” column in Table S1 in the *Supplementary Information* document). While the cell is operated with *I* ≠ 0 *A*, coulomb counting is used to estimate the SoC (see Equation (2)).

2 $$SoC(t)=So{C}_{start}+\Delta So{C}_{chg/dischg}(t)=So{C}_{start}+\frac{\Delta Q(t)}{{C}_{remaining,usable,lastCU,RT,chg/dischg}}$$

The remaining usable charging and discharging capacities determined in the last CU at RT are used in Equation (2). In charging processes, the determined *charging* capacity is considered. For discharging or in dynamic operation (during EIS, pulse pattern measurement, or while applying the driving profiles), the estimated *discharging* capacity of the last CU is used.

While the cell is charged or discharged, the OCV is estimated using the average of an SoC-based and a voltage-based approach. The SoC-based OCV is estimated with the estimated SoC and the OCV-SoC LUT determined for charging or discharging (“chg”/“dischg” columns in Table S1 in the *Supplementary Information* document). For the voltage-based OCV estimation, the measured terminal voltage minus the measured cell current multiplied by the estimated cell resistance is used. For this resistance, the 10 ms pulse resistance determined in the initial current pulse during the pulse pattern measurements at different SoCs (10/30/50/70/90%) and at RT or OT is used as a baseline. The RT or OT pulse resistances are selected depending on the intended cell temperature. Linear interpolation is used to determine the actual resistance value for an SoC between 10 and 90%, and the values of the 10 or 90% measurements are selected outside of this SoC window. The OCV is capped by the measured terminal voltage if the estimated OCV would otherwise be greater than the measured voltage while charging or smaller than the measured voltage while discharging.

After a rest period of 4 minutes and 40 seconds (i.e., 20 seconds before the earliest start of the subsequent charging or discharging procedure), the OCV is determined using the filtered cell voltage again. However, for an accurate OCV estimation, the cell would have to relax for multiple hours or even days. After the OCV is updated, the SoC is adjusted using the new OCV and the SoC-OCV LUT. This assures that the voltage-based SoC method that is now in effect again is used for the SoC_end_ value in the EOC dataset 20 seconds later (compare Equation (1)).

**Fig. 14:**
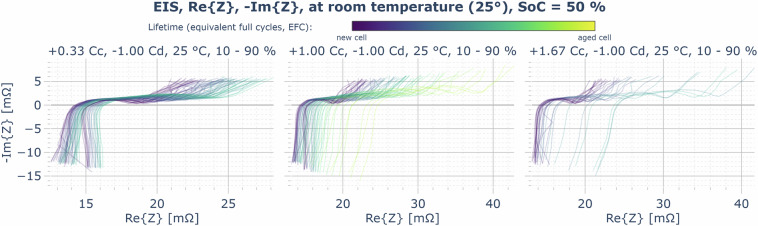
Example plot for the EIS data record: frequency-dependent complex impedance Z, measured in the CUs at 50% SoC and 25°C, comparison of selected cyclic aging cells (aging at 25°C, 10–90% SoC range, at different charging rates: 1/3 C, 1 C, 5/3 C, overlay of the traces of all three cells aging at the respective condition over time — from purple (new) to yellow (highest number of EFCs).

**Fig. 15:**
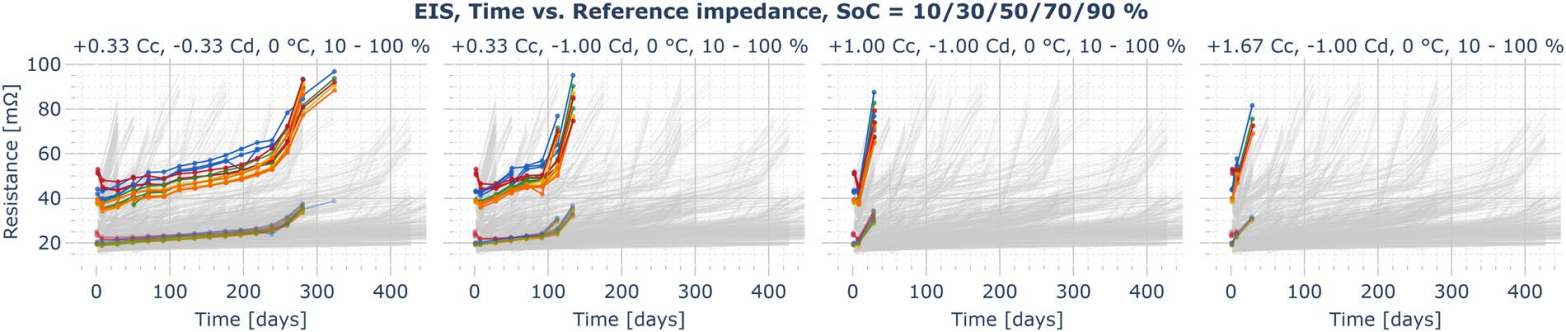
Example plot for the EIS data record: reference impedance Z_ref,0_ (impedance amplitude average of the impedances measured at 0.2083, 0.5, 2.083, and 10 Hz) measured at SoC = 10% (red), 30% (orange), 50% (yellow), 70% (green), 90% (blue) at operating temperature (here: 0°C, opaque/upper colored traces) and room temperature (25°C, semi-transparent/lower colored traces) for cyclic aging cells operated an 0°C, 10−100% SoC range, and various charging- and discharging rates — overlay of the reference impedances for all other cyclic aging cells in gray.

As mentioned in the Data post-processing section, the EIS measurements have a significantly lower quality than measurements that would have been collected using expensive commercial equipment. Nevertheless, clear aging trends can be seen from the EIS measurements (compare Fig. 14) and the derived reference impedance (shown in Fig. 15). If the EIS data is used, for example, to fit an equivalent circuit model (ECM), the data points might need additional filtering. For example, implausible data points could be removed prior to the usage.

**Fig. 16:**
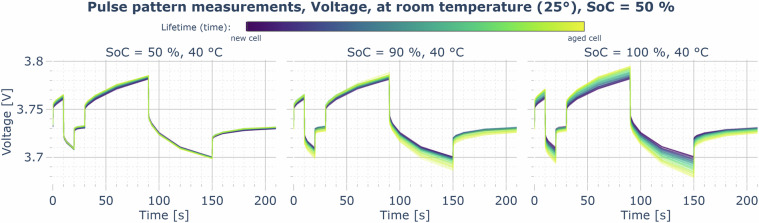
Example plot for the PULSE data record: cell voltage measured at an excitation with a pulse current pattern (+1/3 C,  − 1/3 C, 0 C for 10 s each, then repeated with 60 s each) at 50% SoC and 25°C, comparison of selected calendar aging cells (aging at 40°C, 50/90/100% SoC), overlay of the traces of all three cells aging at the respective condition over time — from dark purple (new) to yellow (highest timestamp).

In contrast, the PULSE measurements (compare Fig. 16) can be used for impedance estimation without further modification, as the terminal voltage and cell current measurement in the time domain, as well as the timing points of the log data, are significantly more precise, and the current controller is very stable and accurate.

Information on issues that could negatively affect data usage (such as data gaps, anomalies, inaccuracies, or NaN values) is summarized in Table S2 in the *Supplementary Information* document.

## Supplementary information

Supplementary Information – Comprehensive battery aging dataset: capacity and impedance fade measurements of a lithium-ion NMC/C-SiO cell

## Data Availability

Example Python scripts facilitating the use of the published battery aging dataset^1^ are freely available on GitHub: https://github.com/energystatusdata/bat-age-data-scripts. The required and optional Python libraries are listed in the “requirements.txt” file in the GitHub repository.
