# Supplementary material for: Comprehensive battery aging dataset: capacity and impedance fade measurements of a lithium-ion NMC/C-SiO cell
Source: Sci Data. 2024 Sep 16;11:1004. doi: 10.1038/s41597-024-03831-x (PMC11405776; doi:10.1038/s41597-024-03831-x)
Supplement: Supplementary file 1 — Supplementary Information – Comprehensive battery aging dataset: capacity and impedance fade measurements of a lithium-ion NMC/C-SiO cell [file 41597_2024_3831_MOESM1_ESM.pdf]

Supplementary Information

Comprehensive battery aging dataset: capacity and impedance fade measurements of a lithium-ion NMC/C-SiO cell

Matthias Luh[1,*] and Dr. Thomas Blank[1,*]

[1]Karlsruhe Institute of Technology (KIT), Institute for Data Processing and Electronics (IPE), Eggenstein-Leopoldshafen, 76344, Germany
[*]corresponding authors: Matthias Luh (matthias.luh@kit.edu), Thomas Blank (thomas.blank@kit.edu)

List of Tables

Table S1 SoC-OCV lookup table used in the experiment . . . 2
Table S2 Overview of data impairments and affected datasets . . . 3

List of Figures

Figure S1 Scheduler states during a CU with a screenshot of the Grafana dashboard . . . 4
Figure S2 Custom battery cycling and data measurement acquisition board used in the experiment . . . 5
Figure S3 Unexpected increase of charging current at colder temperatures and medium to high charging rates . . . 6

SoC [%]	V_{chg} [V]	V_{dischg} [V]	V_{avg} [V]	SoC [%]	V_{chg} [V]	V_{dischg} [V]	V_{avg} [V]	SoC [%]	V_{chg} [V]	V_{dischg} [V]	V_{avg} [V]
0	2.5496	2.5000	2.5000	34	3.6312	3.6050	3.6181	68	3.9236	3.9114	3.9175
1	2.9671	2.6585	2.8128	35	3.6363	3.6131	3.6247	69	3.9313	3.9197	3.9255
2	3.1057	2.8336	2.9696	36	3.6415	3.6204	3.6310	70	3.9393	3.9279	3.9336
3	3.1955	2.9421	3.0688	37	3.6469	3.6271	3.6370	71	3.9475	3.9366	3.9421
4	3.2601	3.0122	3.1362	38	3.6524	3.6338	3.6431	72	3.9562	3.9457	3.9510
5	3.3058	3.0666	3.1862	39	3.6582	3.6400	3.6491	73	3.9655	3.9550	3.9602
6	3.3317	3.1091	3.2204	40	3.6642	3.6467	3.6554	74	3.9748	3.9649	3.9698
7	3.3524	3.1458	3.2491	41	3.6705	3.6533	3.6619	75	3.9848	3.9745	3.9797
8	3.3726	3.1788	3.2757	42	3.6771	3.6603	3.6687	76	3.9952	3.9850	3.9901
9	3.3915	3.2130	3.3022	43	3.6844	3.6674	3.6759	77	4.0055	3.9959	4.0007
10	3.4109	3.2491	3.3300	44	3.6918	3.6749	3.6833	78	4.0160	4.0064	4.0112
11	3.4271	3.2835	3.3553	45	3.6996	3.6829	3.6912	79	4.0264	4.0169	4.0217
12	3.4369	3.3150	3.3759	46	3.7080	3.6911	3.6995	80	4.0364	4.0272	4.0318
13	3.4447	3.3427	3.3937	47	3.7170	3.6995	3.7083	81	4.0460	4.0368	4.0414
14	3.4519	3.3679	3.4099	48	3.7261	3.7089	3.7175	82	4.0552	4.0464	4.0508
15	3.4590	3.3901	3.4246	49	3.7355	3.7179	3.7267	83	4.0633	4.0552	4.0592
16	3.4667	3.4102	3.4385	50	3.7453	3.7271	3.7362	84	4.0702	4.0625	4.0663
17	3.4756	3.4294	3.4525	51	3.7553	3.7368	3.7460	85	4.0757	4.0683	4.0720
18	3.4857	3.4497	3.4677	52	3.7653	3.7464	3.7559	86	4.0800	4.0730	4.0765
19	3.4977	3.4607	3.4792	53	3.7757	3.7560	3.7658	87	4.0832	4.0769	4.0801
20	3.5100	3.4689	3.4894	54	3.7864	3.7658	3.7761	88	4.0861	4.0799	4.0830
21	3.5218	3.4759	3.4988	55	3.7974	3.7753	3.7863	89	4.0891	4.0828	4.0860
22	3.5332	3.4823	3.5077	56	3.8096	3.7846	3.7971	90	4.0923	4.0856	4.0890
23	3.5447	3.4882	3.5165	57	3.8242	3.7947	3.8094	91	4.0958	4.0888	4.0923
24	3.5557	3.4947	3.5252	58	3.8396	3.8043	3.8219	92	4.0997	4.0929	4.0963
25	3.5650	3.5035	3.5342	59	3.8514	3.8144	3.8329	93	4.1048	4.0974	4.1011
26	3.5749	3.5163	3.5456	60	3.8615	3.8259	3.8437	94	4.1108	4.1031	4.1070
27	3.5864	3.5298	3.5581	61	3.8707	3.8380	3.8543	95	4.1183	4.1100	4.1141
28	3.5960	3.5431	3.5696	62	3.8789	3.8505	3.8647	96	4.1279	4.1191	4.1235
29	3.6028	3.5564	3.5796	63	3.8867	3.8637	3.8752	97	4.1406	4.1311	4.1358
30	3.6091	3.5676	3.5883	64	3.8944	3.8746	3.8845	98	4.1565	4.1470	4.1517
31	3.6150	3.5772	3.5961	65	3.9017	3.8846	3.8932	99	4.1765	4.1682	4.1723
32	3.6206	3.5866	3.6036	66	3.9089	3.8942	3.9015	100	4.2000	4.1972	4.2000
33	3.6259	3.5962	3.6111	67	3.9162	3.9031	3.9096				

Table S1. SoC-OCV lookup table used in the experiment. The terminal voltage was measured while charging (V_{chg}) and discharging (V_{dischg}) the cell at 1/20 C to 4.2 and 2.5 V with a cut-off current of 1/100 C. The average of the two values (V_{avg}) is used to determine the SoC of relaxed cells, e.g., in calendar aging. Underlined values were manually edited to match the voltage limits of the cell.

Data records, as named in the paper	CFG				EOC	EIS	PULSE	LOG	LOG_AGE	POOL_LOG	SLAVE_LOG		
**Component**	cell	pool	t.mgmt.	cycler	cell	cell	cell	cell	cell	pool	t.mgmt.	cycler	
**Description** / **Filename prefix**	*cell_cfg*	*pool_cfg*	*slave_cfg_T*	*slave_cfg_S*	*cell_eocv2*	*cell_eisv2*	*cell_plsv2*	*cell_logext*	*cell_log_age*	*pool_log*	*slave_log_T*	*slave_log_S*	**See also chapter...**
calendar aging cells also experience cyclic aging due to the check-ups	i				i	i	i	i	i				Brief evaluation of the measurement results
cyclic & profile aging cells also face calendar aging	i				i	i	i	i	i				Usage Notes
voltage limits ($V = OCV_{new\_cell}(10\%)$) are used instead of charge-based SoC limits (10%) to reach a particular SoC	i				i	i	i	i	i				Parameter set selection
driving profile power might be reduced when the SoC is low	i				i			i	i				Parameter set selection
the hotter of the two cell sensor temperatures is used if $\Delta T > 3\ K$ (usually, the average is used)	i							i	i				Experimental setup
similarly, the hotter of the two pool sensor temperatures is used if $\Delta T > 3\ K$ (usually, the average is used)		i								i			Experimental setup
cell temperature can be significantly higher than the pool temperature due to power losses in the cell	i	i			i	i	i	i	i	i			Experimental setup
implausible coulomb/energy efficiency, remaining usable capacity & SoH estimations are replaced with NaN					i								Data post-processing
SoH definition in dataset differs from the ones commonly used in the literature					i	i							Data quality and inspection
synthetic data in data gaps is marked by an sd_block_id of 0								i					Data quality and inspection
timestamps might be estimated (see timestamp_origin columns)					(x)	(x)	(x)	(x)	(x)	(x)	(x)	(x)	Data post-processing
leakage currents ($\approx 200\ \mu A$) in the cycler slowly drain the cells ($\approx$ 50% SoC/year, noticeable for calendar aging cells)					(x)			(x)	(x)				Test procedure
$\Delta Q/E$, Q/E_{total} are estimated after unexpected cycler resets					(x)			(x)	(x)				Data post-processing
some log measurements might be NaN just after a reboot								(x)		(x)	(x)	(x)	Data quality and inspection
some EIS measurement points and derived Z_{ref} values are NaN when invalid/implausible						(x)							Data quality and inspection
temperature control of the 25°C pool slightly deteriorated after Apr 23, 2023 (slightly cooler, higher temperature variation)	i	i			(x)	(x)	(x)	(x)	(x)	(x)	i		Data quality and inspection
SoC/OCV estimations are not precise (simple algorithms)					(x)	(x)	(x)	(x)	(x)				Usage Notes
SoC/OCV might be particularly inaccurate after unexpected cycler resets during a flash write (e.g., slave 6, Oct 19, 2022)					x	x	x	x	x				Data post-processing
dataset has gaps (cells rest, pools maintain temperature)					(x)			x	x	x	x	x	Data post-processing
low accuracy of EIS measurement						x							Data post-processing
low data quality of unprocessed LOG files (NaN or implausible values)										x	x	x	Data quality and inspection
temperature control of all pools was disabled from Nov 27 to Nov 29, 2022 (ΔT up to 13 K)	i	i			x	x	x	x	x	x	x		Data quality and inspection

Severity:

i	informative (no negative impairment, but worth knowing)
(x)	minor impairment of data
x	significant impairment of data

Component:

cell	individual battery cell
pool	thermally controlled liquid bath in which the cells are placed
cycler	device controlling and measuring the cells
t.mgmt.	thermal management device controlling pool temperatures
slave	either a cycler or a thermal management device

Table S2. Overview of data impairments and affected datasets

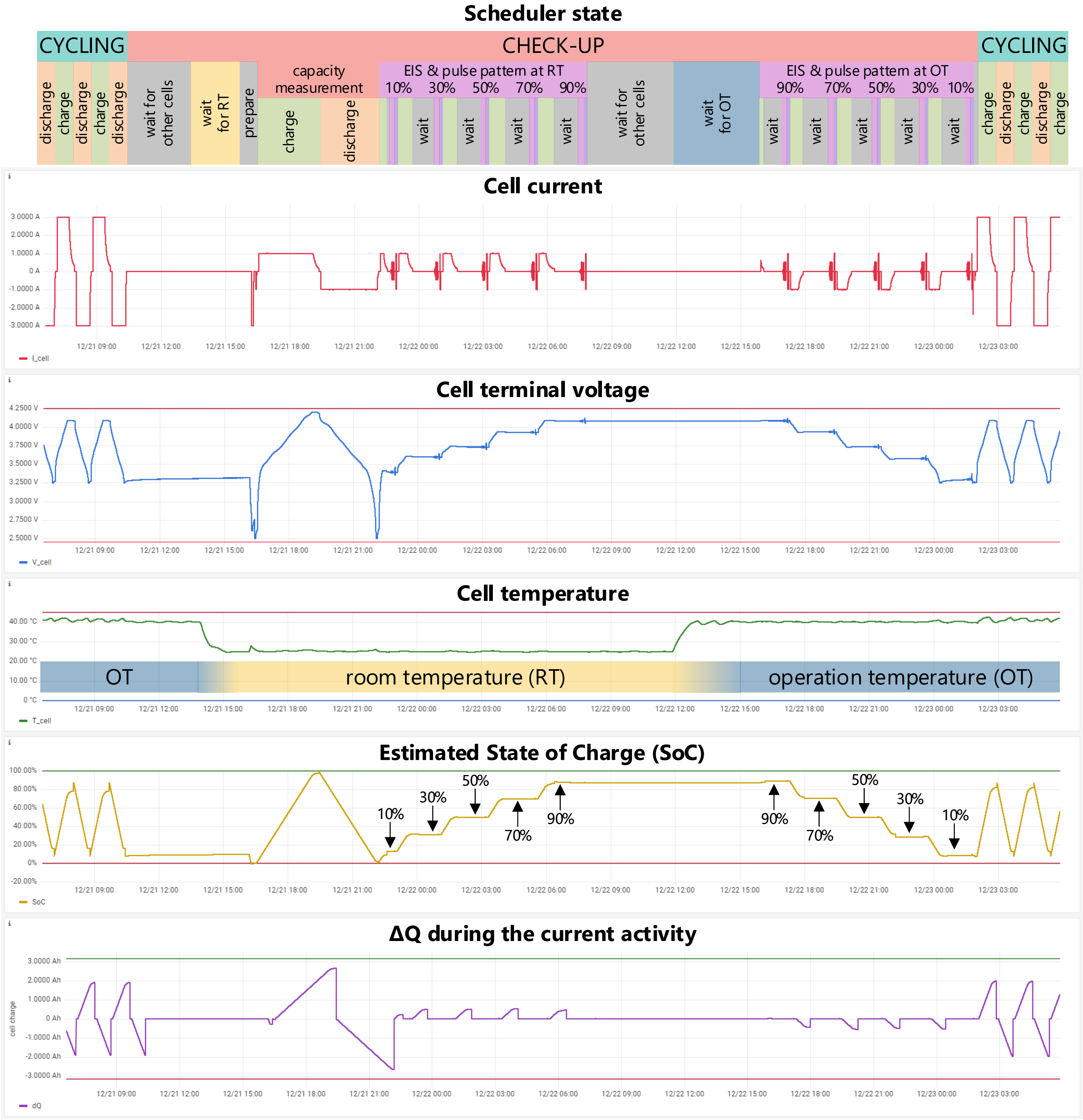

Figure S1. Scheduler states during a CU with a screenshot of the Grafana dashboard showing the corresponding cell current [A], voltage [V], temperature [T], SoC [%], and charge/discharge [Ah] — December 21 to 23, 2022, P063-1, S16:C05

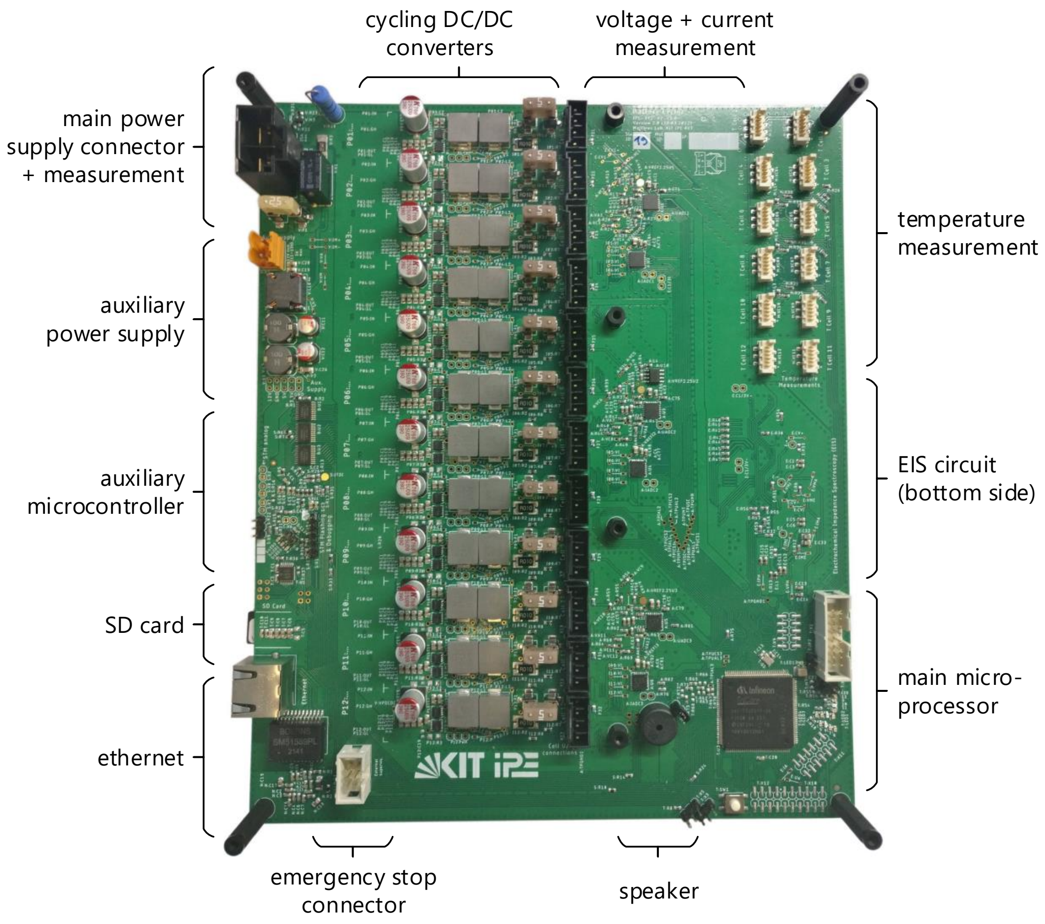

Figure S2. Custom battery cycling and data measurement acquisition board used in the experiment

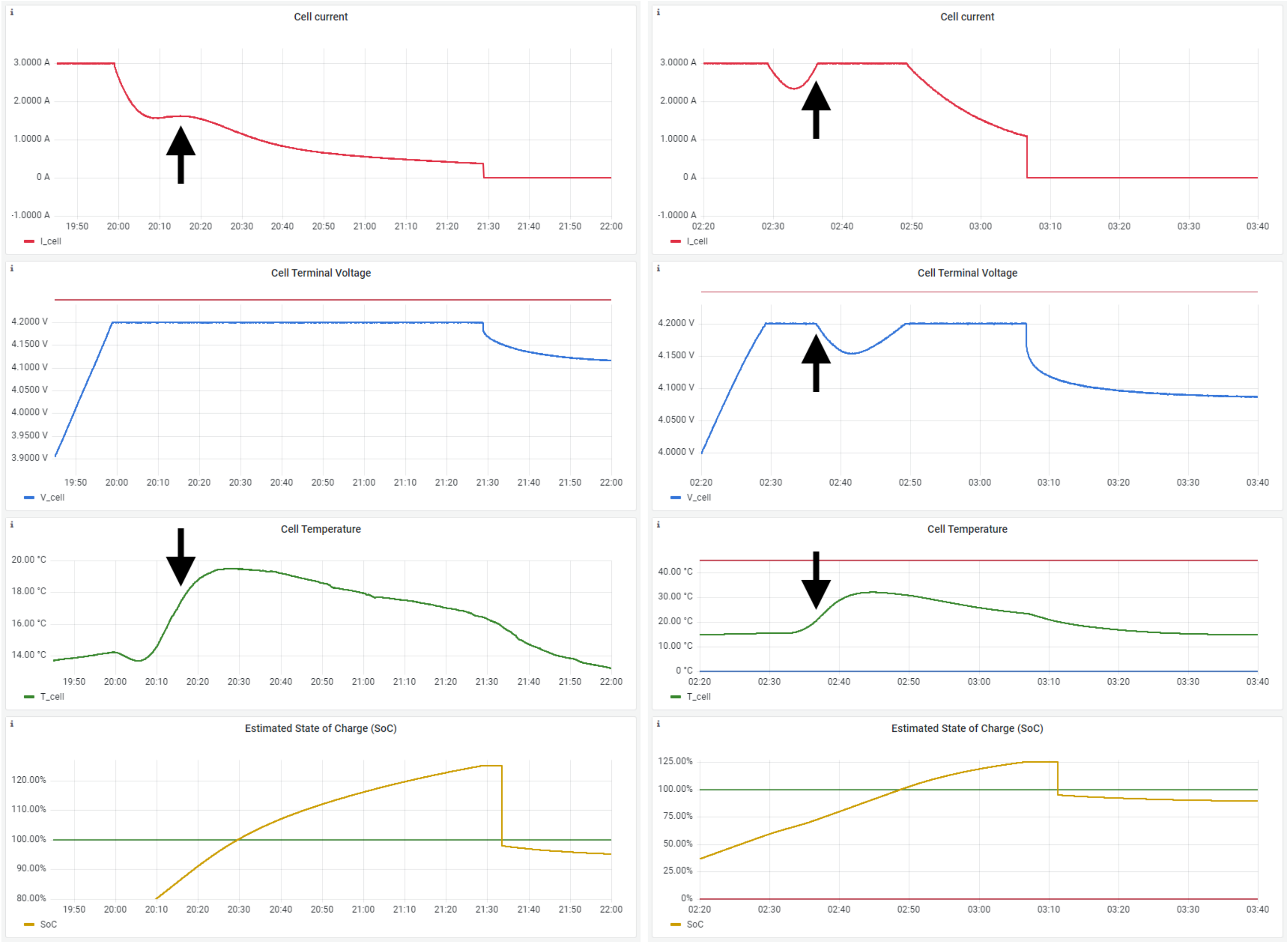

Figure S3. Unexpected increase of charging current at colder temperatures and medium to high charging rates (here: 1.0 C to 4.2 V, 10°C pool temperature) in the CV charging phase (left), which sometimes even causes a second CC phase (right), indicating lithium plating and lithium stripping. From top to bottom: cell current (red), voltage (blue), and temperature (green), estimated SoC (yellow). In both cases, an unusually severe temperature increase was detected even though the cells were liquid-cooled. Both cells were permanently disabled before the end of the charging process since the BMS issued an SoC-based overcharge event — the coulomb-counting-based SoC reached 125%, i.e., the charge transferred into the cell was significantly higher than expected. Left: P035-1, S08:C04, December 2, 2022, from 6:45 PM to 9:00 PM, right: P031-2, S07:C04 November 11, 2022, 01:20 AM to 02:40 AM.
